# M^6^A demethylase FTO-stabilized exosomal circBRCA1 alleviates oxidative stress-induced granulosa cell damage via the miR-642a-5p/FOXO1 axis

**DOI:** 10.1186/s12951-024-02583-5

**Published:** 2024-06-25

**Authors:** Xiaolan Zhu, Wenxin Li, Minjun Lu, Junyu Shang, Jiamin Zhou, Li Lin, Yueqin Liu, Jie Xing, Mengxue Zhang, Shijie Zhao, Jingjing Lu, Xuyan Shi

**Affiliations:** 1 Reproductive Medicine Center, The Fourth Affiliated Hospital of Jiangsu University, No. 20 Zhengdong Road, Zhenjiang, 212001 Jiangsu China; 2 Department of Central Laboratory, The Fourth Affiliated Hospital of Jiangsu University, Zhenjiang, China; 3 Reproductive Sciences Institute, Jiangsu University, Zhenjiang, China

**Keywords:** Premature ovarian insufficiency (POI), HucMSCs-Exs, circBRCA1, N6-methyladenosine (m^6^A), FTO

## Abstract

**Background:**

Premature ovarian insufficiency (POI) is an important cause of female infertility and seriously impacts the physical and psychological health of patients. Human umbilical cord mesenchymal stem cell-derived exosomes (HucMSCs-Exs, H-Exs) have exhibited protective effects on ovarian function with unclear mechanisms.

**Methods:**

A comprehensive analysis of the Gene Expression Omnibus (GEO) database were used to identify POI-associated circRNAs and miRNAs. The relationship between HucMSC-derived exosomal circBRCA1/miR-642a-5p/FOXO1 axis and POI was examined by RT-qPCR, Western blotting, reactive oxygen species (ROS) staining, senescence-associated β-gal (SA-β-gal) staining, JC-1 staining, TEM, oxygen consumption rate (OCR) measurements and ATP assay in *vivo* and in *vitro*. RT-qPCR detected the expression of circBRCA1 in GCs and serum of patients with normal ovarian reserve function (n = 50) and patients with POI (n = 50); then, the correlation of circBRCA1 with ovarian reserve function indexes was analyzed.

**Results:**

Herein, we found that circBRCA1 was decreased in the serum and ovarian granulosa cells (GCs) of patients with POI and was associated with decreased ovarian reserve. H-Exs improved the disorder of the estrous cycles and reproductive hormone levels, reduced the number of atretic follicles, and alleviated the apoptosis and senescence of GCs in rats with POI. Moreover, H-Exs mitigated mitochondrial damage and reversed the reduced circBRCA1 expression induced by oxidative stress in GCs. Mechanistically, FTO served as an eraser to increase the stability and expression of circBRCA1 by mediating the m^6^A demethylation of circBRCA1, and exosomal circBRCA1 sponged miR-642a-5p to block its interaction with FOXO1. CircBRCA1 insufficiency aggravated mitochondrial dysfunction, mimicking FTO or FOXO1 depletion effects, which was counteracted by miR-642a-5p inhibition.

**Conclusion:**

H-Exs secreted circBRCA1 regulated by m^6^A modification, directly sponged miR-642a-5p to upregulate FOXO1, resisted oxidative stress injuries in GCs and protected ovarian function in rats with POI. Exosomal circBRCA1 supplementation may be a general prospect for the prevention and treatment of POI.

**Graphical Abstract:**

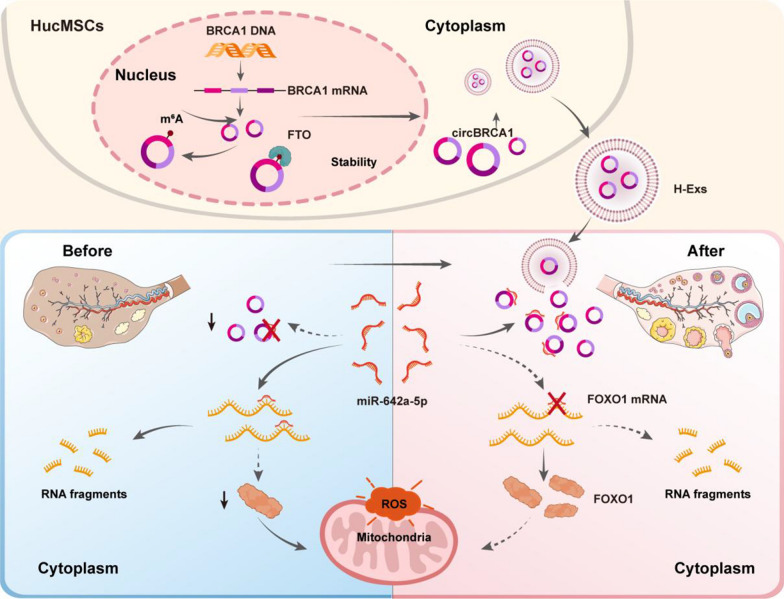

**Supplementary Information:**

The online version contains supplementary material available at 10.1186/s12951-024-02583-5.

## Background

Premature ovarian insufficiency (POI) is a disease characterized by early menopause before 40 years of age, accompanied by an elevation of follicle-stimulating hormone (FSH ≥ 25 IU/L on two occasions over 4 weeks apart), and ultimately leads to female infertility [1]. POI is one of the most common reproductive endocrine disorders and affects 1–2% of women of childbearing age [2]. Ovarian granulosa cells (GCs) dysfunction triggered by reactive oxygen species (ROS) exposure is an important causal factor for POI [3]. Mitochondria act as intracellular organelles for producing ROS, and disrupted mitochondrial function induces the senescence and even apoptosis of GCs, thereby contributing to the occurrence of POI [4, 5].

The current conventional clinical treatment methods for POI are characterized by several side effects, and most do not effectively restore the physiological functions of the ovaries [6]. Thus, novel treatment options for mitochondrial disorder-linked POI require further exploration. Transplantation of human umbilical cord mesenchymal stem cell-derived exosomes (HucMSCs-Exs, H-Exs) could prevent the formation of oxidative stress-induced ROS and DNA damage and thus comprehensively rescue GCs damage by transporting therapeutic proteins, nucleic acids and lipids. This method is considered a promising regenerative medicine approach and has received substantial attention in the management of POI with high efficacy and low immunogenicity and toxicity [7, 8]. Recently, noncoding RNAs (ncRNAs), including microRNAs, long ncRNAs and circRNAs carried by H-Exs, have begun to be explored in ovaries [9–11].

CircRNAs, a novel type of ncRNA characterized by a covalently closed loop without a 5′ end cap or 3′ poly(A) tail [12], are highly conserved and characterized by tissue-specific expression patterns [13] and mainly remain in the cytoplasm and function as miRNA sponges [14]. The loop structures without free ends make circRNAs more stable than other RNAs, and thus, circRNAs are ideal biomarkers and therapeutic targets for multiple diseases [15]. Recently, differentially expressed circRNAs have been discovered in human GCs during maternal aging, suggesting the involvement of circRNAs in the maintenance of ovarian function [16]. However, the role of exosomal circRNAs in the regulation of POI pathogenesis remains largely unknown.

N^6^-methyladenosine (m^6^A) modification is the most abundant internal modification of both coding and noncoding RNA polymerase II transcripts with major impacts on their dynamic regulation [17]. Reportedly, m^6^A controls the post-transcriptional regulation of circRNAs, including nuclear export, backsplicing, and translation, by recruiting specific m^6^A reader/eraser proteins [18]. Abnormally elevated levels of m^6^A in the ovaries have been implicated in POI [19, 20]. The “writers” of m^6^A are mainly composed of the METTL3/METTL14 methyltransferase complex and its cofactor WTAP [21]. The process of demethylation is primarily mediated by the methylase fat mass and obesity-associated (FTO) and ALKBH5 [22, 23]. The most recent studies found that FTO acted as an "eraser" of m^6^A methylation and decreased apoptosis in GCs by regulating the expression of BNIP3 [24] and retarding FOS-dependent ovarian aging [25], suggesting that FTO may play a non-negligible role in the regulation of ovarian function. Based on available published data, a clear view of m^6^A modification in circRNAs has been established, and m^6^A modification enhanced the transcriptome stability of circMDK, which partially accounts for the significant upregulation of circMDK [26]. In addition, circNSUN2 m^6^A modification facilitated cytoplasmic export and stabilized HMGA2 [27].

In the present study, we screened and identified the expression and functions of hsa_circ_0043949 (circBRCA1) derived from BRCA1 and examined the detailed mechanism of exosomal circBRCA1 in the regulation of POI pathology. Functional experiments revealed that FTO acted as a novel transcriptional activator of circBRCA1 via m^6^A demethylation, which upregulated circBRCA1 expression. Moreover, exosomal circBRCA1 led to an increase in the target gene FOXO1 through competitive binding to miR-642a-5p, thereby repairing oxidative damage in GCs. Our findings highlight that exosomal circBRCA1 secreted from HucMSCs improves ovarian function and ameliorates mitochondrial damage, leading to the development of specific therapies for POI.

## Methods

### Patients and samples

50 patients with POI (POI group) who were treated with in vitro fertilization or intracytoplasmic sperm injection and embryo transfer (IVF/ICSI-ET) at the Reproductive Center of the Fourth Affiliated Hospital of Jiangsu University (Zhenjiang Maternal and Child Health Hospital) were selected from June 2021 to July 2023; 50 patients with normal ovarian reserve function (NC group) who underwent IVF/ICSI-ET due to male and/or tubal factors were selected as controls during the same period. The age and BMI of the 2 groups were compared, and the differences were not statistically significant (P > 0.05), and were comparable.

All patients with POI in this study were clearly diagnosed by attending physicians and above. The collection of patients' GCs and serum samples (taken on an empty stomach in the early morning of the 2nd-3rd day of menstruation) was approved by the Ethics Committee of The Fourth Affiliated Hospital of Jiangsu University (Zhenjiang Maternal and Child Health Hospital), which was in accordance with the ethical requirements, and all subjects participated voluntarily and signed a written informed consent. The basic information of the patients is shown in Table S1.

### Cell culture and transfection

The human granulosa cell line KGNs and the human embryonic kidney cell line 293 T were purchased from the Chinese Academy of Sciences Cell Bank (Shanghai, China), cultured in F12/DMEM medium (Gibco, USA) with 10% FBS (Gibco, USA). Human umbilical cord tissues were obtained from the Fourth Affiliated Hospital of Jiangsu University, and each parturient woman signed an informed consent form in advance. HucMSCs were isolated as previously reported [28] and cultured in α-MEM (Gibco, USA) with 10% FBS (Gibco, USA). All cells maintained in a humidified incubator at 37 °C with 5% CO_2_.

The si-circBRCA1, si-FOXO1, si-FTO, si-ALKBH5, miR-642a-5p inhibitor, biotin-miR-642a-5p and the corresponding controls (si-NC, miR-NC, biotin-miR-NC) were purchased from Genepharma (Suzhou, China). We performed transfection using Lipofectamine 2000 reagent (Invitrogen, Carlsbad, USA) according to the manufacturer's instructions.

### Isolation and characterization of H-Exs

When the degree of HucMSCs at passage 3 to 5 fusion reached 80% ~ 90%, FBS were replaced with Ex-free FBS, and the HucMSCs continued to be cultured for 48 h. The cell supernatant was centrifuged at 4 °C, 2000 g for 20 min to remove cellular debris and concentrated using ultrafiltration device (UFC900396, Millipore, USA) at 4 °C, 2000 g for 30 min. H-Exs were isolated using total exosome isolation kit (ECS; Umibio, Shanghai, China). Dissolved the H-Exs in PBS (Gibco, USA) and store them at −80 ℃ for subsequent experiments. Purified H-Exs was labeled with PKH26 (Sigma-Aldrich, USA) for Exs uptake assays.

We observed the size and structure of H-Exs with a transmission electron microscope (TEM) and nanoparticle tracking analysis (NTA), and tested the protein marker CD9 and CD63 of H-Exs by Western Blot analysis.

### Fish

The Cy3-labeled hsa_circ_00043949 (circBRCA1) probe was designed and synthesized by GenePharma (Suzhou, China). The sequence of the probe is 5′-CCTCTGACTTCAAAATCATG TGTGCCAAGGGTGAATGATG-3′. The RNA FISH kit was purchased by GenePharma (Suzhou, China). KGNs were grown on round coverslips and processed according to the kit's instruction. FISH probes were diluted (1:50), denatured, balanced and added to KGNs at 37 °C overnight. After hybridization, cells were stained with DAPI for 15 min at room temperature. Finally, the results were observed with fluorescence microscope (Leica Microsystems, Mannheim, Germany).

### Nucleus-cytoplasm extraction

Follow the manufacturer's instructions, the Nuclear and Cytoplasmic Protein Extraction Kit (Beyotime, Shanghai, China) was used to isolate the nucleus and cytoplasm of KGNs. RNA was extracted from the nucleus and cytoplasm respectively, and the expression level of circBRCA1 in the nucleus and cytoplasm was detected by RT-qPCR according to the above method. GAPDH was used as the cytoplasmic control and U6 was used as the nuclear control.

### ActD and DAA treatment

After 0 h, 6 h, 12 h, and 24 h of KGNs with 2 mg/mL actinomycin D (Sigma, USA), total RNA is extracted for PT-qPCR to assess the stability of circBRCA1 and its linear gene BRCA1. After 48 h of KGNs with DAA treatment, total RNA was extracted for PT-qPCR to detect differential expression of circBRCA1.

### Western blot

Samples of cells and ovarian tissues were lysed by using radioimmunoprecipitation assay (RIPA) lysis buffer (Solarbio, Beijin, China) containing protease inhibitor (Solarbio, Beijin, China). Determined the protein concentration of the samples, added 5 × Lodding buffer (Beyotime, Shanghai, China) and boiled in the water bath for 5 min. Electrophoresis was performed on 8% or 10% sodium dodecyl sulfate polyacrylamide gel (SDS-PAGE) and then transferred onto polyvinylidene fluoride (PVDF) membranes (Millipore, USA). The membranes were blocked with 5% nonfat milk for 2 h, and then incubated with anti-FOXO1 (Abcepta, Suzhou, China, 1:500), anti-SOD_2_ (Abcam, USA, 1:1000), anti-P21 (CST, USA, 1:1000) or anti-GAPDH (Proteintech, Wuhan, China, 1:10000) overnight at 4 °C. After the anti-IgG (Biosharp, China, 1:10000) were incubated at room temperature for 1.5 h, the signals were detected by the enhanced chemiluminescence reagent kit (ECL; Vazyme, Nanjing, China) and alyzed by Image J software.

### RNA and gDNA extraction and quantitative real‑time polymerase chain reaction (RT-qPCR)

According to the instructions, tissues and cells were lysed in TRIzol reagent (Ambion, USA) to extract total RNA. Genomic DNA (gDNA) was extracted from KGNs by the Genomic DNA kit (Tiangen, Beijing, China). Reverse transcriptased mRNA by HiScript II Q RT SuperMix (Vazyme, Nanjing, China). MiRNA Universal SYBR qPCR Master Mix (Vazyme, Nanjing, China) was used for miRNA cDNA synthesis. Quantitative reverse transcription polymerase chain reaction (PCR) was performed using ChamQ Universal SYBR qPCR Master Mix (Vazyme, Nanjing, China). Glyceraldehyde 3-phosphate dehydrogenase (GAPDH) or U6 was utilized as an endogenous reference. All primers were designed and synthesized by Shanghai Sangon Biotech and listed in Table S2.

### ROS assay

The Reactive Oxygen Species Assay Kit (Beyotime, Shanghai, China) is used to evaluate the level of ROS in KGNs and rat ovarian tissues. Applied DCFH-DA probes to sections of KGNs or ovarian tissues in different experimental groups, the samples were incubated at 37 °C for 30 min in a light shielded humidifying incubator and washed with PBS for 1–2 times. The nuclei were incubated with DAPI (Solarbio, Beijin, China) at room temperature in dark condition for 10 min, cleaned with PBS for 1–2 times, and the images were taken immediately under fluorescence microscope (Leica Microsystems, Mannheim, Germany). Image J software is used to analyze fluorescence intensity.

### Mitochondrial membrane potential assay

The Mitochondrial membrane potential assay kit with JC-1 (Beyotime, Shanghai, China) was used to detect the mitochondrial membrane potential of KGNs. JC-1 probes (1 mg/L) were loaded at 37 °C for 20 min. After washed with PBS for 1–2 times, immediately taken pictures with fluorescence microscope (Leica Microsystems, Mannheim, Germany), Image J software is used to analyze fluorescence intensity.

### Senescence-associated β-gal assay

The Senescence β-Galactosidase Staining Kit (Beyotime, Shanghai, China) reflected the aging of KGNs, and SA-β-gal staining was conducted according to the manufacturer’s instructions. The cells were cultured in six-well plates, fixed with 1 ml of fixative solution per well for 15 min at room temperature. The cells were washed twice with PBS and incubated overnight with freshly prepared SA-β-gal staining solution in the aforementioned incubator. The number of senescent cells was evaluated by counting the number of blue-stained cells under the light microscope.For rat ovarian tissue section staining, the frozen slices were fixed with fixative solution for 15 min and stained overnight with SA-β-gal staining solution in the incubator.Then, the frozen slices were restained with eoxin dye and observed under the light microscope (Leica Microsystems, Mannheim, Germany).

### Oxygen consumption rate (OCR) measurements and ATP levels

OCR measurements were performed using a Seahorse Bioscience XFp Extracellular Flux Analyzer instrument (Agilent Technologies AG, Basel, Switzerland). Oxygen consumption was measured every 10 min and the following injections were performed after every three measurements: (1) 1 mM oligomycin, (2) 1 mM FCCP, and (3) 2 mM rotenone and 2 mM antimycin A. The results were normalized to cell number quantified by DNA content.

ATP levels were determined using the ATP Assay Kit (Beyotime, China) according to the manufacturer's instructions.

### Methylated RNA immunoprecipitation (MeRIP) assay

The RNA Immunoprecipitation (RIP) Kit (BersinBio, Guangzhou, China) was used to validate the interaction between circBRCA1 and m^6^A. KGNs lysates were incubated with the prepared magnetic beads along with anti-m^6^A (Abcam, USA, 4 μl) or anti-IgG (BersinBio, Guangzhou, China, 4 μl) at 4 °C. Collected and washed magnetic beads. RT-qPCR was employed to test circBRCA1 expression.

### Establishment of the model of POI and treatment with H-Exs

Five-week-old Sprague Dawley (SD) female rats were purchased from the Animal Experiment Center of Jiangsu University. All animal experimental protocols were approved by the Institutional Animal Care and Use Committee (IACUC) of the Fourth Affiliated Hospital of Jiangsu University.

To establish the POI rat model, rats were injected intraperitoneally with 8 mg/kg cyclophosphamide (CTX) on the first day. Thereafter, rats were injected intraperitoneally with 2 mg/kg CTX daily for 13 days. A normal control group (NC) was injected with normal saline instead of CTX [29]. On the fifteenth day of model establishment, POI rats were randomly divided into four groups, which received PBS, Exs, H-Exs-si-circBRCA1 and H-Exs-si-FTO by tail vein injection.

### Assessment of rat estrous cycles

Vaginal smears of rats were obtained at 9:00 a.m. each day to observe the estrous cycle in each group. The normal estrous cycle in rats consists of the following four consecutive phases: proestrus, estrus, metestrus, and diestruss, which were identified according to the presence or absence of cornified epithelium, nucleated epithelial cells, and leukocytes [29] Experiments included rats with at least two consecutive normal estrous cycles.

### Ovarian morphology analysis and follicle counts

Six pairs of ovaries were collected after 2 weeks of H-Exs treatment, photographed and weighed to record ovarian size and ovarian index. Fresh ovarian tissues were fixed with 4% paraformaldehyde for one day, dehydrated, paraffin embedded and cut into 4 μm sections for the following experiments. Sections were stained with hematoxylin and eosin (HE). Ovarian morphology was observed using Pathology Image Scanner (Pannoramic MIDI, Hungary), and the number of follicles was counted according to the previous method [30].

### Immunohistochemistry

Sections were deparaffinized and rehydrated and heated in a microwave oven to repair the antigen. Then, sections were incubated with primary antibodies, including FOXO1 (Abcepta, China, 1:50), overnight at 4 °C. Subsequently, sections were incubated with a horseradish peroxidase-conjugated secondary antibody (Biosharp, China, 1:10000) was incubated at room temperature in the dark. Counterstained the sections with hematoxylin (Biosharp, China), and imaged with the Pathology Image Scanner (Pannoramic MIDI, Hungary).

### Immunofluorescence Staining

Sections were permeated by 0.3% Triton X-100, blocked with 10% FBS (Gibco, USA), and incubated in the presence of primary antibodies, including FSHR (Proteintech, China, 1:200), Ki67 (Abcam, USA, 1:3000), and FOXO1 (Abcepta, China, 1:50) at 4 °C overnight, followed by incubation with a horseradish peroxidase-conjugated secondary antibody (Biosharp, China, 1:10000) for 1 h at 37 °C in the dark. The sections were counterstained with DAPI and imaged using the Pathology Image Scanner (Pannoramic MIDI, Hungary).

### Elisa

An enzyme-linked immunosorbent assay kit (ImmunoWay, USA) was used to detect AMH, FSH, LH and E_2_. The serum samples were collected and incubated with ninety-six-well plates with antibodies at room temperature for 2 h. The serum hormone levels were measured by the microplate reader (Bio-Rad, USA).

### Statistical analysis

The experimental data were representative of three independent experiments at least, and the GraphPad Prism software (version 9.0 for Windows) was used for calculations and generated the statistical graph. The differences between two groups were analyzed using two-tailed t-tests. One-way analysis of variance (ANOVA) was used for the multiple group comparisons. When P < 0.05, it was considered statistically significant in this study. Pearson correlation and linear regression analysis were performed to evaluate the correlation between circBRCA1 and clinical ovarian reserve indicators in the POI group (n = 50) and all participants (n = 100). *P < 0.05, **P < 0.01, ***P < 0.001, ****P < 0.0001.

## Results

### H-Exs alleviated oxidative damage by improving mitochondrial function in KGNs

**Fig. 1:**
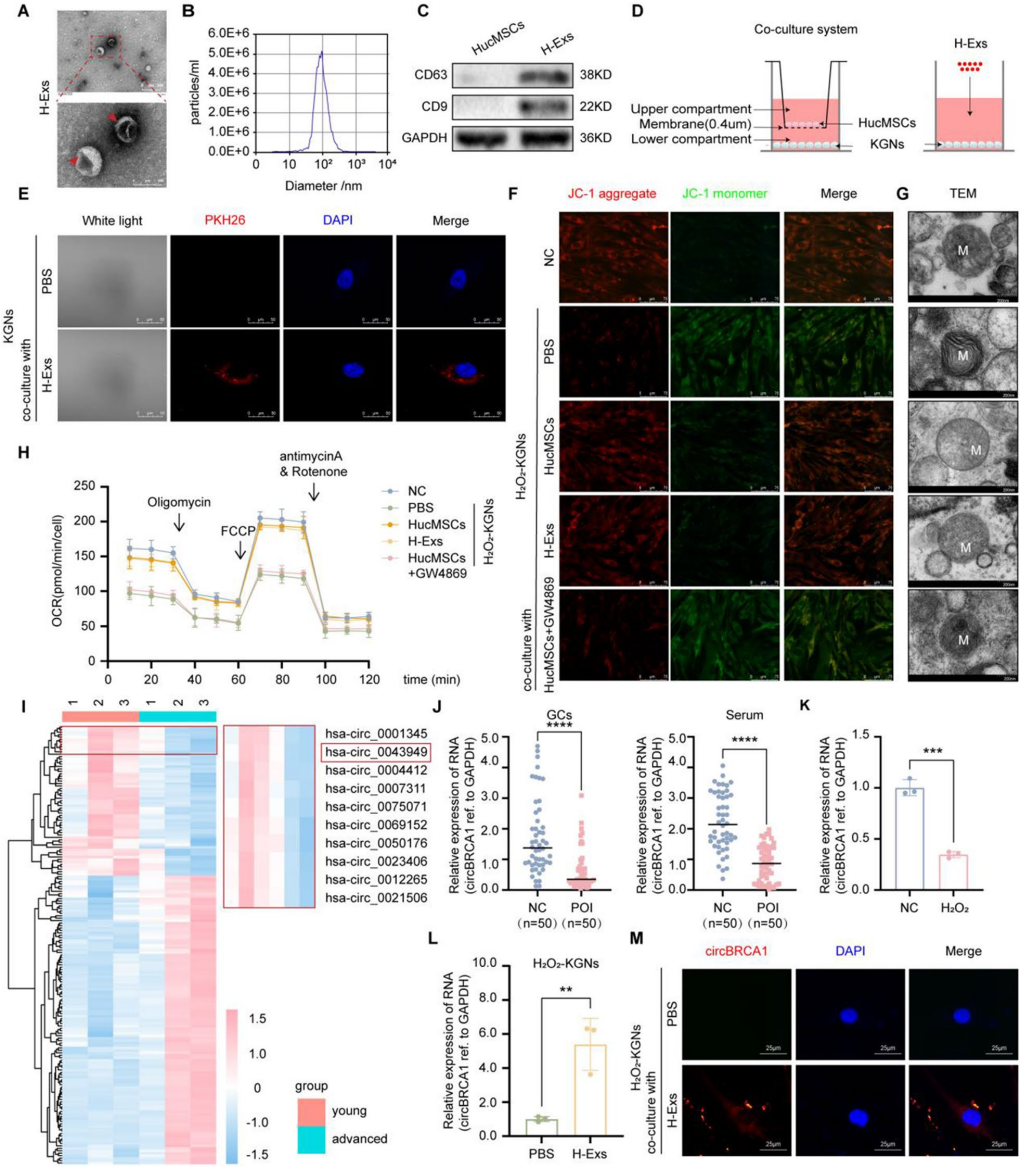
CircBRCA1 is significantly upregulated in oxidatively damaged KGNs treated with H-Exs. **A** Scanning of H-Exs using TEM. (scale bar = 100 nm) **B** The size range of the H-Exs checked by NTA. **C** Western blot analysis of H-Ex-related markers CD9 and CD63. **D** Schematic diagram of HucMSCs or H-Exs co-culture with KGNs. **E** Internalization of PKH-26-labeled-H-Exs was analyzed in H_2_O_2_-KGNs co-cultured with H-Exs. Red: PKH26-H-Exs staining; Blue: nuclear staining. (scale bar = 50 µm) **F** JC-1 staining was used to detect the changes of MMP. (scale bar = 75 µm) **G** Scanning the mitochondria of KGNs using TEM. (scale bar = 200 nm) **H** Oxygen consumption rate of KGNs. **I** Hierarchical clustering of diferentially expressed circRNAs in samples of GCs from women with advanced age (AA, ≥ 38 years) and young age (YA, ≤ 30 years). **J** Relative expression levels of circBRCA1 in GCs and serum of patients with normal ovarian function (NC, n = 50) and POI patients (POI, n = 50) were determined by RT-qPCR. **K** Relative expression levels of circBRCA1 in KGNs and H_2_O_2_-KGNs were determined by RT-qPCR. **L** Relative expression levels of circBRCA1 in KGNs after HucMSCs/H-Exs transplantation were determined by RT-qPCR. **M** FISH assay was used to illustrate that CY3-marked-circBRCA1 in H-Exs was internalized by H_2_O_2_-KGNs

Previously, we demonstrated that H-Exs were able to alleviate GCs senescence by reducing ROS damage [31]. Considering that mitochondria are the first sensors of senescence and target organs of ROS production [2, 5, 32], we hypothesize that Ex-mediated antisenescence effects may be associated with improvements in the mitochondrial function of GCs. To illustrate this, we isolated and characterized H-Exs and evaluated the effect of H-Exs in the repair of oxidative damage-induced mitochondrial dysfunction in KGNs (the in vitro cell line of GCs). As revealed by transmission electron microscopy (TEM), nanoparticle tracking analysis (NTA) and Western blot analysis, H-Exs were vesicles with a spherical shape and a bilayer membrane structure (Fig. 1A), approximately 100 nm in diameter (Fig. 1B), and highly expressed the exosome-surface marker proteins CD9 and CD63 (Fig. 1C).

In the coculture systems (Fig. 1D), PKH26-marked-H-Exs were absorbed by KGNs (Fig. 1E). Subsequently, we constructed in vitro models of oxidative injury using H_2_O_2_ (H_2_O_2_-KGNs), which was mainly characterized by elevated ROS, senescence marker P16, P21 and SA-β-gal activity and decreased levels of the antioxidant enzymes Gpx and SOD_2_ in KGNs (Supplementary Fig. 1A-G).

Notably, we found that H_2_O_2_ treatment hindered the mitochondrial activity of KGNs, as evidenced by decreased mitochondrial membrane potential (MMP), OCR and ATP levels and increased abnormal mitochondria (Fig. 1F–H and Supplementary Fig. 1H, I), indicating that H_2_O_2_ treatment had a significant inhibitory effect on the mitochondrial respiratory chain. However, HucMSCs/H-Exs transplantation not only ameliorated oxidative stress-induced cellular senescence but also repaired mitochondrial function (Fig. 1F–H and Supplementary Fig. 1A-I). Nevertheless, GW4869 addition (for inhibiting neutral sphingomyelinase and blocking H-Exs release) impaired HucMSC-mediated repair function (Fig. 1F–H and Supplementary Fig. 1A-I), suggesting that HucMSCs reduced oxidative damage in H_2_O_2_-KGNs by delivering H-Exs.

### CircBRCA1 is significantly upregulated in oxidatively damaged KGNs treated with H-Exs

Recently, the H-Exs transfer of ncRNAs (noncoding RNAs) was regarded as a novel and important mechanism of genetic exchange between cells, and the role of circRNAs in POI is receiving increasing attention. To search for circRNAs that may be associated with POIs, we analyzed data from the GEO dataset (GSE97193), including RNA-seq data of circRNAs in GCs with three advanced age (AA, > 38 years) and three young (YA, < 30 years) women undergoing IVF/ICSI-ET, and found that a total of 179 circRNAs, 61 downregulated and 118 upregulated circRNAs, were differentially expressed in GCs. The heatmap was used to illustrate the differentially expressed pattern of circRNAs (Fig. 1I). Among the top 30 most significantly downregulated circRNAs, by RT-qPCR, we verified that only hsa_circ_0043949 was significantly decreased in GCs and serum of patients with POI and in H_2_O_2_-KGNs (Fig. 1J, K), while this molecule was obviously increased in H_2_O_2_-KGNs after coculture with H-Exs (Fig. 1L). Consistently, hsa_circ_0043949 was enriched in HucMSCs/H-Exs (Supplementary Fig. 1J), and its host parental gene was associated with mitochondrial function [33–35], which was selected for our next studies. Hsa_circ_0043949 was further referred to as circBRCA1 because it consists of exons of BRCA1. As expected, FISH analysis revealed that Cy3-marked circBRCA1 in H-Exs was internalized by H_2_O_2_-KGNs (Fig. 1M).

### Characterization and identification of circBRCA1

**Fig. 2:**
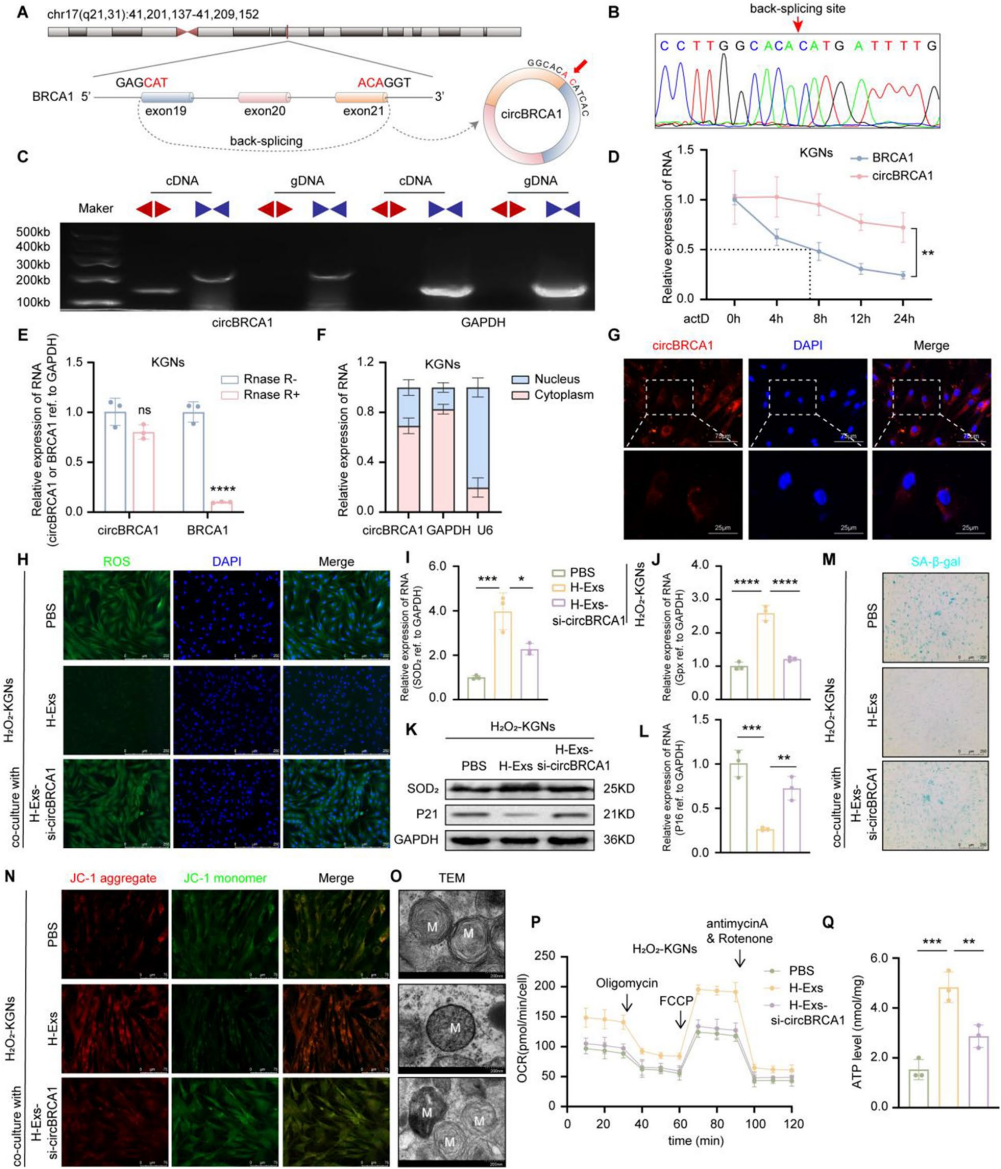
H-Ex-released circBRCA1 attenuates mitochondrial damage of KGNs. **A** CircBRCA1 is cyclized from exons 19–21 of the BRCA1 gene. **B** The back-splicing junction site of circBRCA1 was verified by Sanger sequencing. **C** Existence of circBRCA1 in KGNs was verified by agarose gel electrophoresis. **D** After actD treatment, the relative expression levels of circBRCA1 and linear BRCA1 mRNA were determined by RT-qPCR. **E** After RNase R treatment, the relative expression levels of circBRCA1 and linear BRCA1 mRNA were determined by RT-qPCR. **F** Cytoplasmic and nuclear separation assay was used to detect the subcellular localization of circBRCA1 in KGNs. **G** FISH assay was used to illustrate the subcellular localization of circBRCA1 in KGNs. **H** The ROS levels were detected by DCFH-DA staining. (scale bar = 50 µm) **I**-**J** Relative expression levels of SOD_2_ and Gpx were determined by RT-qPCR. **K** Western blot analysis revealed the expression levels of SOD_2_ and P21 in KGNs. **L** Relative expression levels of P16 were determined by RT-qPCR. **M** SA-β-gal staining. (scale bar = 100 µm) **N** JC-1 staining was used to detect the changes of MMP. (scale bar = 15 µm) **O** Scanning the mitochondria of KGNs using TEM. (scale bar = 200 nm) **P** Oxygen consumption rate of KGNs. **Q** Statistical analysis of ATP level.

The 213-bp-long circBRCA1 is formed by circularization of exons 19–21 of the BRCA1 gene, which is located at chromosome chr17(q21,31):41,201,137–41,209,152 (Fig. 2A). Then, we used Sanger sequencing to confirm whether exons 19–21 of the BRCA1 gene were backspliced to form a closed loop structure (Fig. 2B). Because head-to-tail splicing could be the consequence of either transsplicing or genomic rearrangements, we designed special convergent primers for circBRCA1 and divergent primers to amplify circBRCA1 by using cDNA and gDNA, respectively. CircBRCA1 was readily amplified by the divergent primers in cDNA but not in gDNA (Fig. 2C). CircBRCA1 was markedly more stable than BRCA1 mRNA following transcriptional inhibition with actinomycin D (ActD) (Fig. 2D). Although linear BRCA1 mRNA was readily degraded by RNase R, circBRCA1 was resistant to this digestion (Fig. 2E). The above results confirmed that circBRCA1 was an abundant, circular and stable transcript and can be an ideal candidate molecule for diagnosis. Furthermore, a nuclear mass separation assay (Fig. 2F) and RNA-FISH analysis (Fig. 2G) for the subcellular distribution of circBRCA1 in KGNs revealed that circBRCA1 was predominantly localized in the cytoplasm.

### H-Ex-released circBRCA1 attenuates mitochondrial damage in KGNs

Given the biofunction of the host gene BRCA1, circBRCA1 may be involved in the repair of mitochondrial damage [33–35]. To clarify this, we transfected small interfering RNAs (siRNAs) into HucMSCs to silence circBRCA1 and collected H-Exs (H-Exs-si-circBRCA1). H-Exs-si-circBRCA1 transplantation significantly reduced the uptake of circBRCA1 but not BRCA1 mRNA by H_2_O_2_-KGNs (Supplementary Fig. 2A). Subsequently, we observed that circBRCA1 KD dramatically reduced the protective effect of H-Exs against oxidative damage. As shown, the H-Exs-si-circBRCA1 group had elevated ROS accumulation (Fig. 2H and Supplementary Fig. 2B) and reduced Gpx and SOD_2_ levels (Fig. 2I–K and Supplementary Fig. 2C), suggesting that circBRCA1 KD attenuated the repair of oxidative damage in H-Exs. Moreover, P16 and P21 levels (Fig. 2K, L) and SA-β-gal activity (Fig. 2M and Supplementary Fig. 2D) were higher in the H-Exs-si-circBRCA1 group than in the H-Exs group, demonstrating that circBRCA1 KD reduced the antisenescent effect of H-Exs.

Next, we further confirmed the effect of exosomal circBRCA1 on mitochondrial activity. CircBRCA1 KD significantly decreased MMP, OCR and ATP levels and increased the number of aberrant mitochondria (Fig. 2N–Q and Supplementary Fig. 2E). These data illustrated that circBRCA1 deficiency leads to mitochondrial dysfunction. To verify that linear BRCA1 mRNA plays a role in H-Exs treatment of POI, we examined the expression of linear BRCA1 mRNA and found that it was barely expressed in H-Exs (Supplementary Fig. 2F). Overall, these results suggest that exosomal circBRCA1 is the key factor by which H-Exs improve mitochondrial function and repair oxidative damage in KGNs.

### CircBRCA1 improves the mitochondrial function of KGNs through the miR-642a-5p/FOXO1 *axis*

**Fig. 3:**
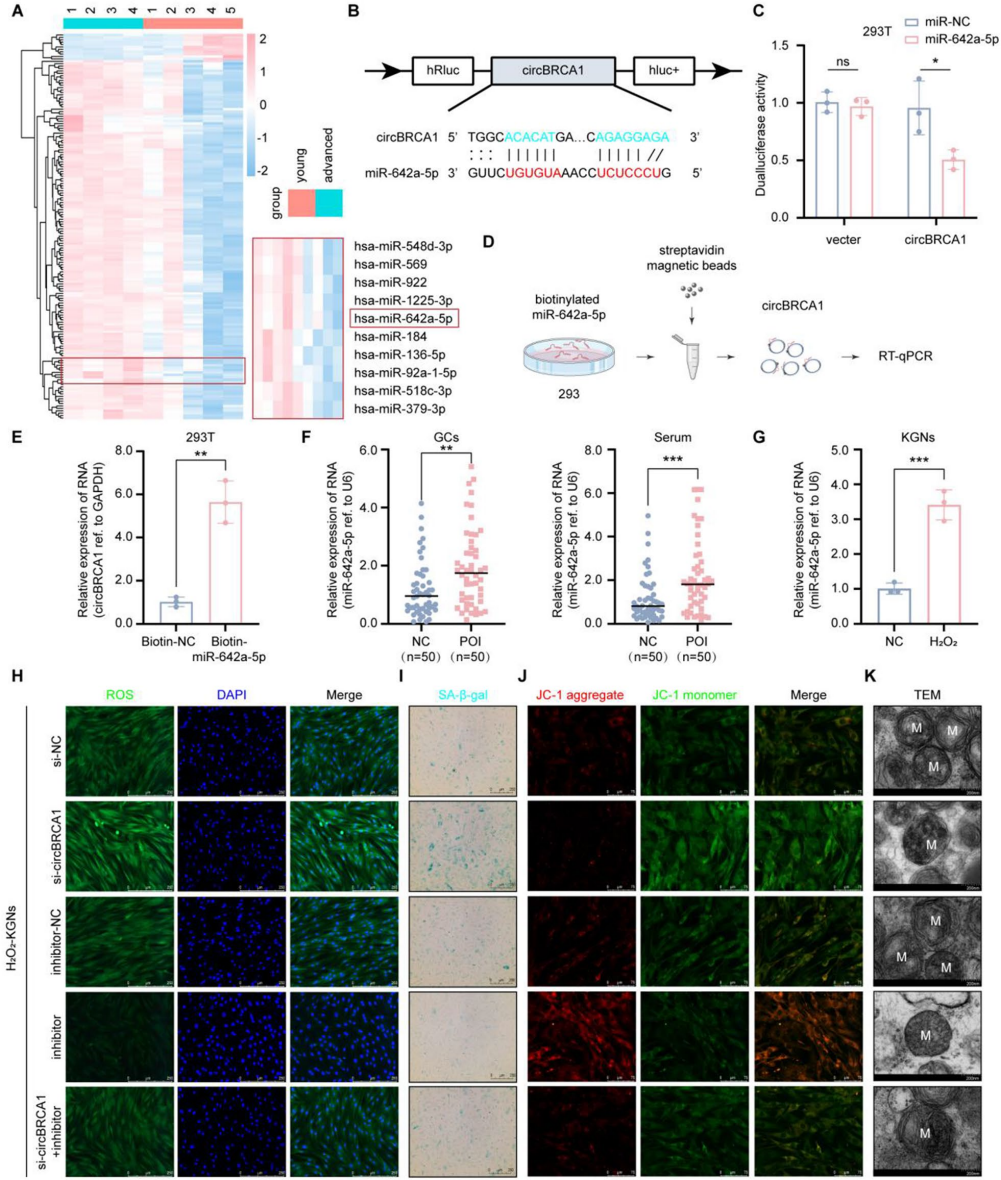
CircBRCA1 acts as a sponge for miR-642a-5p. **A** Hierarchical clustering of diferentially expressed miRNAs in samples of follicular fluid from women with advanced age (AA, ≥ 36 years) and young age (YA, ≤ 36 years). **B** Schematic representation of circBRCA1 binding to miR-642a-5p. **C** Dualluciferase reporter assay using vectors containing overexpression of circBRCA1 in 293 T cells transfected with negative control (miR-NC) or miR-642a-5p miRNA mimics. **D** Schematic representation of pull-down assay with biotinylated miR-642a-5p. **E** Enrichment of circBRCA1 in 293 T cells after pull-down assay with biotinylated miR-642a-5p. **F** Relative expression levels of miR-642a-5p in GCs and serum of patients with normal ovarian function (NC, n = 50) and POI patients (POI, n = 50) were determined by RT-qPCR. **G** Relative expression levels of miR-642a-5p in KGNs and H_2_O_2_-KGNs. **H** The ROS levels were detected by DCFH-DA staining. (scale bar = 50 µm) **I** SA-β-gal staining. (scale bar = 100 µm) **J** JC-1 staining was used to detect the changes of MMP. (scale bar = 75 µm) **K** Scanning the mitochondria of KGNs using TEM. (scale bar = 200 nm)

Given that circBRCA1 is mainly localized in the cytoplasm and is highly stable, we further investigated whether circBRCA1 exerts its biological functions by sponging miRNAs. To identify abnormally expressed miRNAs in patients with POI, we analyzed the data of the GEO dataset (GSE63737), including miRNA RNA-seq data of follicular fluid from 4 advanced-age (> 36 years old) women and 5 young (< 36 years old) women. The results showed that a total of 174 miRNAs, 11 downregulated and 163 upregulated miRNAs, were differentially expressed in follicular fluid. In parallel, we predicted 9 candidate miRNAs with binding sites for circBRCA1 sequences using the miRanda database (http://www.microrna.org/microrna/home.do) (Supplementary Fig. 3A). Of these, only miR-642a-5p was present in the 163 upregulated miRNAs (Fig. 3A). To confirm the binding between circBRCA1 and miR-642a-5p, we used vectors overexpressing circBRCA1 in 293 T cells transfected with negative control (miR-NC) or miR-642a-5p mimics. The dual-luciferase reporter assay showed that miR-642a-5p mimics significantly decreased circBRCA1 luciferase activity (Fig. 3B, C). Then, we designed a 3′ terminal-biotinylated-miR-642-5p probe. As shown in Fig. 3D, circBRCA1 was significantly enriched in the pulled down material compared to the control group (Fig. 3E). Collectively, these results reveal that miR-642a-5p could directly bind to circBRCA1. Not surprisingly, miR-642a-5p was abundant in the serum and GCs of patients with POI (Fig. 3F), as well as in H_2_O_2_-KGNs (Fig. 3G). These data indicate that circBRCA1 acts as a sponge for miR-642a-5p.

To illustrate whether miR-642a-5p is involved in circBRCA1-mediated regulation of mitochondrial function, we transfected miR-642a-5p inhibitor into H_2_O_2_-KGNs. As shown, the miR-642a-5p inhibitor reduced ROS accumulation, P16 and P21 levels and SA-β-gal activity but elevated Gpx and SOD_2_ levels (Fig. 3H, I and Supplementary Fig. 3B-G), supporting that the miR-642a-5p inhibitor repaired ROS-induced cellular senescence. In addition, the miR-642a-5p inhibitor elevated MMP (Fig. 3J and Supplementary Fig. 3H), OCR and ATP levels (Supplementary Fig. 3I, J) and reduced the number of aberrant mitochondria (Fig. 3K), which indicated that the miR-642a-5p inhibitor attenuated mitochondrial dysfunction. As expected, the miR-642a-5p inhibitor rescued the biological effects caused by si-circBRCA1 (Fig. 3H–K and Supplementary Fig. 3B-J). These functional experiments confirmed that circBRCA1 improved mitochondrial function by targeting miR-642a-5p to prevent oxidative damage-induced cellular senescence.

**Fig. 4:**
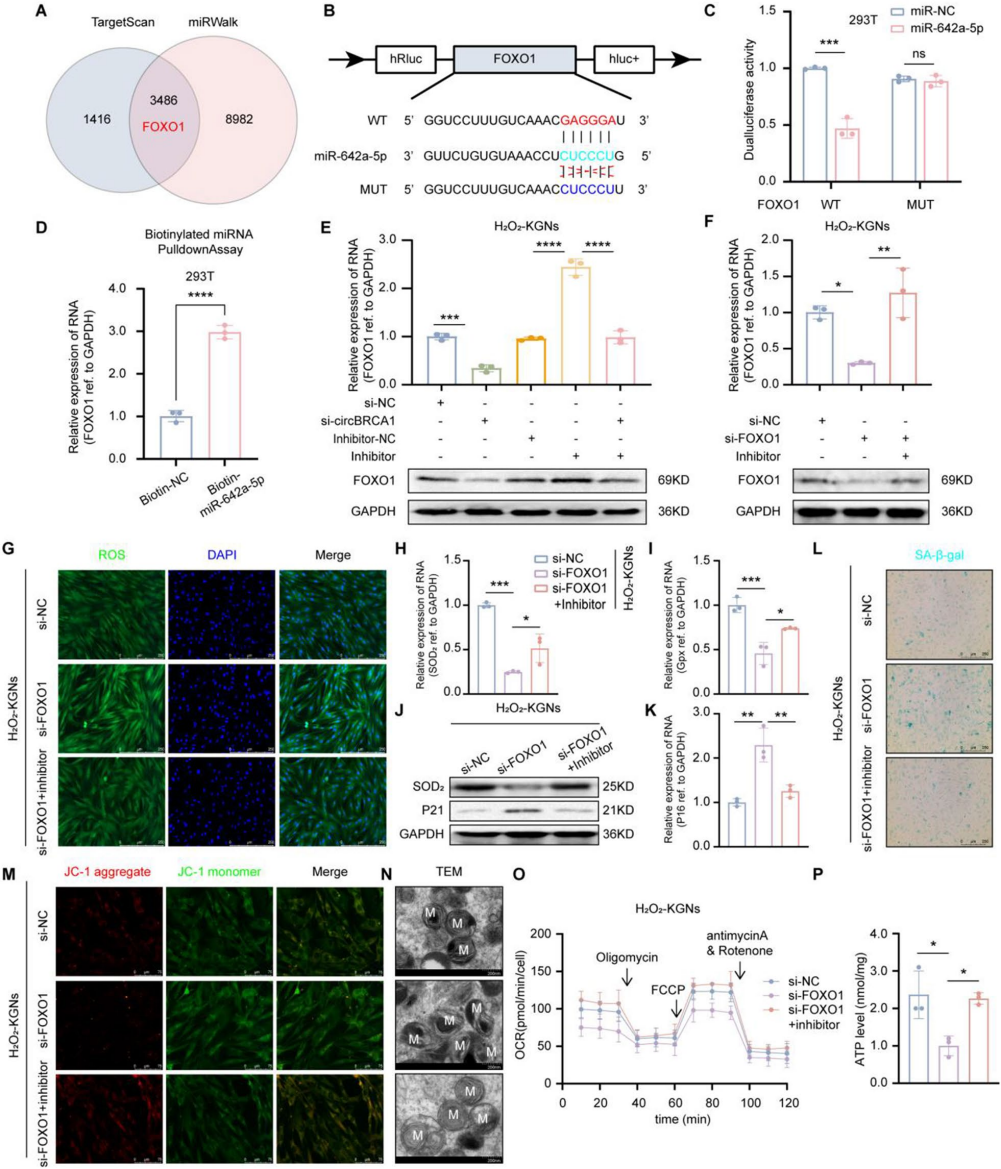
CircBRCA1 upregulates FOXO1 expression by sponging miR-642a-5p. **A** The schematic drawing of the screening procedure for miR-642a-5p candidate targets. **B** Schematic representation of FOXO1 binding to miR-642a-5p. **C** Dualluciferase reporter assay using FOXO1 3′-UTR constructs containing wild-type or mutant seed sequences in 293 T cells transfected with negative control (NC) or miR-642a-5p miRNA mimics. **D** Enrichment of FOXO1 in 293 T cells after pull-down assay with biotinylated miR-642a-5p. **E**–**F** Relative expression levels of FOXO1 were determined by RT-qPCR and western blot analysis. **G** The ROS levels were detected by DCFH-DA staining. (scale bar = 50 µm) **H**–**I** Relative expression levels of SOD_2_ and Gpx were determined by RT-qPCR. **J** Western blot analysis revealed the expression levels of SOD_2_ and P21 in KGNs. **K** Relative expression levels of P16 were determined by RT-qPCR. **L** SA-β-gal staining. (scale bar = 100 µm) **M** JC-1 staining was used to detect the changes of MMP. (scale bar = 15 µm) **N** Scanning the mitochondria of KGNs using TEM. (scale bar = 200 nm) **O** Oxygen consumption rate of KGNs. **P** Statistical analysis of ATP level

To further explore the downstream target genes of circBRCA1/miR-642a-5p in POI, we predicted the potential targets of miR-642a-5p by TargetScan (http://www.targetscan.org) and miRWalk (http://mirwalk.umm.uni-heidelberg.de/). Given the role of FOXO1 in oxidative stress regulation and cellular senescence, we finally selected FOXO1 as a potential signaling pathway downstream of circBRCA1/miR-642a-5p (Fig. 4A). To confirm the molecular binding of miR-642a-5p to FOXO1, we constructed wild-type and mutant sequences of FOXO1 and performed dual-luciferase reporter assays in 293 T cells (Fig. 4B). The results showed that miR-642a-5p reduced the luciferase activity of the wild-type reporter gene, while no significant change was found using the mutant reporter gene (Fig. 4C). The RNA pulldown assay demonstrated that FOXO1 was significantly enriched in the fraction captured by miR-642a-5p compared to that of the mimic-NC group (Fig. 4D). In addition, transfection of H2O2-KGNs with the miR-642a-5p inhibitor significantly increased FOXO1 levels, and this effect was reversed by circBRCA1 silencing (Fig. 4E). Furthermore, FOXO1 was found to be decreased in GCs and serum of patients with POI, as well as in H_2_O_2_-KGNs (Supplementary Fig. 4A, B). In summary, FOXO1 is a functional downstream target of the circBRCA1/miR-642a-5p axis.

To verify the function of FOXO1, we transfected si-FOXO1 into H_2_O_2_-KGNs cells and found that FOXO1 levels were significantly decreased (Fig. 4F). As before, FOXO1 KD elevated ROS accumulation and P16 and P21 levels and SA-β-gal activity but reduced Gpx and SOD_2_ levels (Fig. 4G–L and Supplementary Fig. 4E-G), confirming that FOXO1 KD exacerbated ROS-induced cellular senescence. Furthermore, FOXO1 KD decreased MMP (Fig. 4M and Supplementary Fig. 4H), OCR and ATP levels (Fig. 4O, P) and increased the number of aberrant mitochondria (Fig. 4N), indicating aggravation of mitochondrial dysfunction. As expected, the miR-642a-5p inhibitor reversed these biological effects induced by si-FOXO1 (Fig. 4G–P and Supplementary Fig. 4E-H). In summary, we revealed a novel role for H-Exs-circBRCA1 in preventing oxidative damage-induced mitochondrial dysfunction and cellular senescence by acting as a ceRNA of miR-642a-5p, resulting in the upregulation of FOXO1.

### FTO-mediated M^6^A demethylation facilitates circBRCA1 stability and expression

**Fig. 5:**
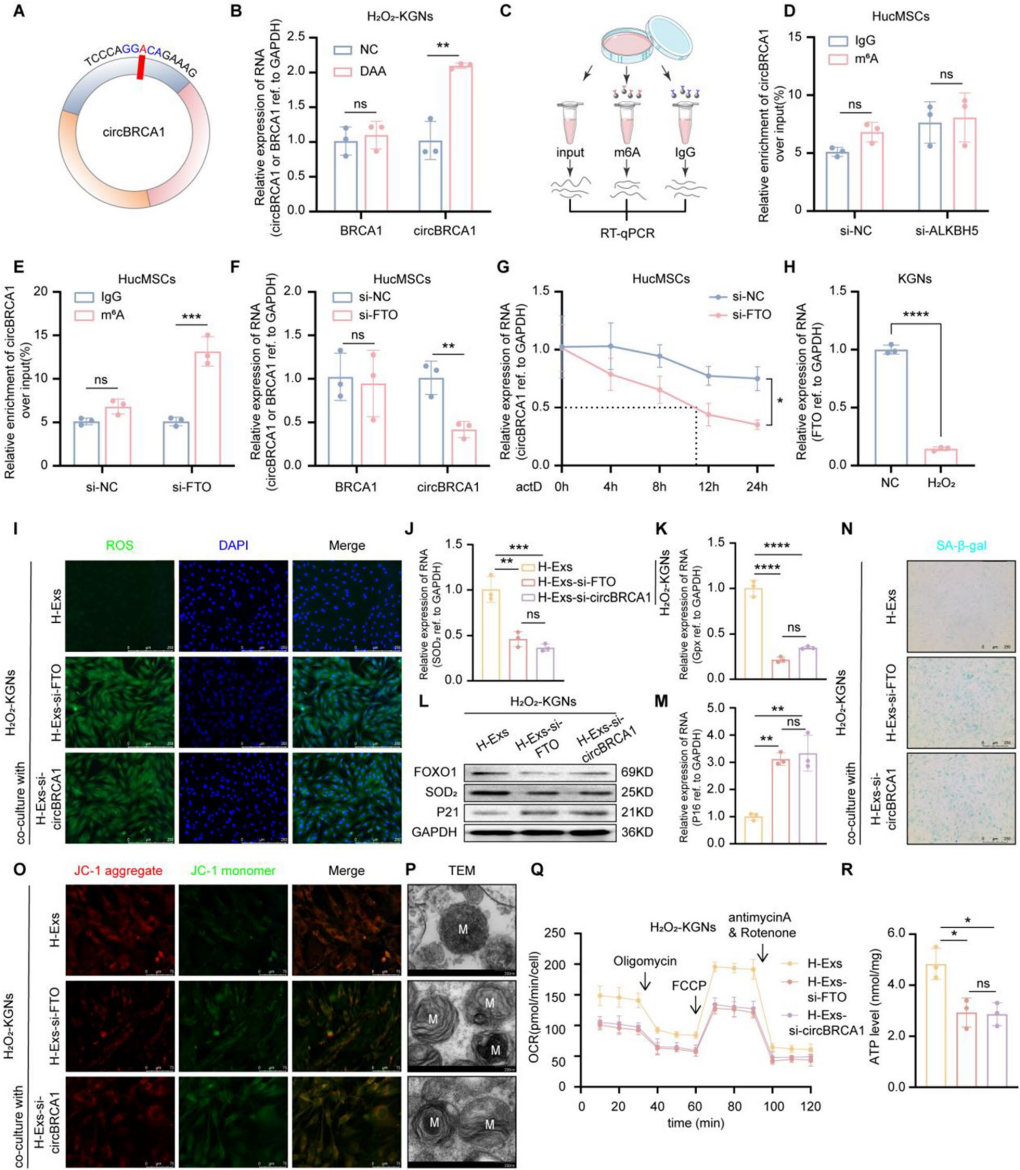
FTO-mediated m^6^A demethylation regulates circBRCA1 stability and expression. **A** Prediction of m^6^a methylation sites on circBRCA1. **B** After treatment with the methylation inhibitor DAA, relative expression levels of circBRCA1and BRCA1 were detected by RT-qPCR. **C** Schematic diagram of Methylated RIP assay. **D**–**E** M^6^A RIP assay was performed to detect the enrichment rate of circBRCA1 in HucMSCs after si-ALKBH5 and si-FTO. **F** Relative expression levels of circBRCA1 and BRCA1 were determined by RT-qPCR. **G** After actinomycin D treatment, the relative expression levels of circBRCA1 were determined by RT-qPCR. **H** Relative expression levels of FTO in KGNs and H_2_O_2_-KGNs were determined by RT-qPCR. **I** The ROS levels were detected by DCFH-DA staining. (scale bar = 50 µm) **J**–**K** Relative expression levels of SOD_2_ and Gpx were determined by RT-qPCR. **L** Western blot analysis revealed the expression levels of SOD_2_ and P21 in KGNs. **M** Relative expression levels of P16 were determined by RT-qPCR. **N** SA-β-gal staining. (scale bar = 100 µm) **O** JC-1 staining was used to detect the changes of MMP. (scale bar = 15 µm) **P** Scanning the mitochondria of KGNs using TEM. (scale bar = 200 nm) **Q** Oxygen consumption rate of KGNs. **R** Statistical analysis of ATP level

M^6^A modification plays a pivotal role in post-transcriptional regulation and the biogenesis of circRNAs. To verify whether the enrichment of circBRCA1 in H-Exs is related to m^6^A methylation, we used SRAMP (http://www.cuilab.cn/sramp) to predict potential m^6^A sites in circBRCA1 and found an m^6^A site in exon 19 (Fig. 5A and Supplementary Fig. 5A). To explore the effects of methylation on the expression of circBRCA1, we treated H_2_O_2_-KGNs with the methylation inhibitor 3-deazaadenosine and detected an increase in circBRCA1 but not BRCA1 mRNA (Fig. 5B).

Knocking down the common m^6^A demethylase FTO but not ALKBH5 decreased the m^6^A methylation level of circBRCA1 (Fig. 5C–E). As expected, FTO KD reduced circBRCA1 levels (but not BRCA1 levels) and stability in HucMSCs (Fig. 5F, G). Consistently, FTO was significantly downregulated in GCs and serum of patients with POI (Supplementary Fig. 5D) and in H_2_O_2_-KGNs (Fig. 5H), indicating that the demethylation-regulating effects of FTO on circBRCA1 may be equally present in other cells, such as KGNs.

Furthermore, the effects of FTO on circBRCA1 were evaluated. We silenced FTO in HucMSCs and collected H-Exs (H-Exs-si-FTO) and found that FTO was significantly decreased (Supplementary Fig. 5D). Similar to H-Exs-si-circBRCA1, H-Exs-si-FTO reduced H-Exs-circBRCA1 levels (Supplementary Fig. 5E), which indicated that FTO KD inhibits the secretion of H-Exs-circBRCA1. Moreover, H-Exs-si-FTO increased ROS accumulation and P16 and P21 levels and SA-β-gal activity but reduced Gpx, SOD_2_, and FOXO1 levels (Fig. 5I–N and Supplementary Fig. 5F-I). In addition, H-Exs-si-FTO decreased MMP (Fig. 5O and Supplementary Fig. 5 J), OCR and ATP levels (Fig. 4Q, R) and increased the number of aberrant mitochondria (Fig. 5P), demonstrating that FTO KD, similar to circBRCA1 KD, reduced the repair of mitochondrial damage by H-Exs. However, simultaneous transfection of si-FTO and si-circBRCA did not further exacerbate ROS-induced cellular senescence and mitochondrial damage (Supplementary Fig. 6A-C). In summary, the above results show that FTO-mediated m^6^A demethylation regulates circBRCA1 stability and expression, which in turn improves mitochondrial function in KGNs.

### *The therapeutic efficacy of exosomal circBRCA1 in treating POI *in vivo

**Fig. 6:**
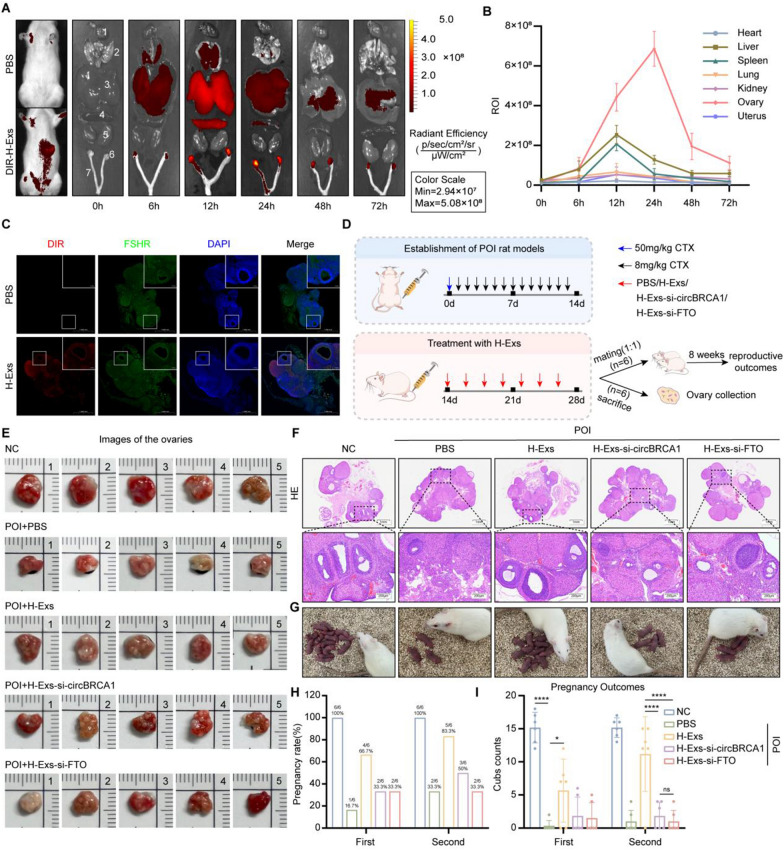
The therapeutic efficacy of exosomal circBRCA1 in treating POI in vivo. **A** Representative images of in vivo fluorescence signals of DiR-labeled H-Exs in major organs (heart, liver, spleen, lungs, and kidney) and ovaries at 0, 6, 12, 24, 48, and 72 h after tail vein injection. **B** Quantitative statistics of fluorescence signal intensity in different organs. **C** Transplanted H-Exs were tracked in rat ovaries. Red: H-Exs labeled with DIR; Blue: nuclear staining. (scale bar = 50 µm). **D** Schematic design for establishment of POI rat models and H-Exs treatment. **E** Ovaries and uterus of rats in each experimental group at the end of study. **F** H&E staining of ovarian sections. (scale bar = 200 µm) **G** Live births photos of each groups. **H** Pregnancy rate at 4 weeks and 8 weeks after treatment. **I** The number of offspring in the five groups at 4 weeks and 8 weeks after treatment

Next, we established POI rat models and tracked the distribution of H-Exs in vivo. DIR-labeled H-Exs were injected into rats with POI via the tail vein. The strongest fluorescent signals of DIR-labeled H-Exs were detected at the site of ovarian damage in rats with POI compared to other organs, and the accumulation of the in vivo optical imaging system was time dependent, with the strongest fluorescent signals being detected at 24 h post-injection (Fig. 6A, B). The colocalization of FSHR-labeled GCs and DIR-labeled H-Exs in the rat ovaries demonstrated the targeting of H-Exs to GCs (Fig. 6C).

Then, the therapeutic potential of exosomal circBRCA1 was evaluated (Fig. 6D). As shown, H-Exs injection for 2 weeks positively affected body weight (Supplementary Fig. 7A), serum hormones (Supplementary Fig. 7B) and the estrous cycle (Supplementary Fig. 7C, D), as well as ovarian size (Supplementary Fig. 7E), ovarian index (Supplementary Fig. 8A) and follicle count in rats with POI (Fig. 6F and Supplementary Fig. 8B), whereas there was almost no significant improvement in the H-Exs-si-circBRCA1 and H-Exs-si-FTO groups, suggesting that circBRCA1 KD and FTO KD impeded the effect of H-Exs in promoting the recovery of ovarian function in rats with POI. In addition, pathological sections showed that H-Exs treatment did not exhibit a negative effect on other organs (Supplementary Fig. 8C), indicating the safety and feasibility of H-Exs therapy. Four and 8 weeks after H-Exs treatment, we assessed the fertility outcomes and found that the pregnancy rate and the number of offspring in two consecutive births were lower in the H-Exs-si-circBRCA1 and H-Exs-si-FTO groups than in the H-Exs group, indicating that circBRCA1 or FTO KD inhibits the potential therapeutic effect of H-Exs in ameliorating CTX-induced fertility loss. Thus, exosomal circBRCA1 promotes the recovery of ovarian function and reproductive ability in rats with POI.

### *Exosomal circBRCA1 regulates GCs mitochondrial dysfunction and senescence through the miR-642a-5p/FOXO1 axis *in vivo

**Fig. 7:**
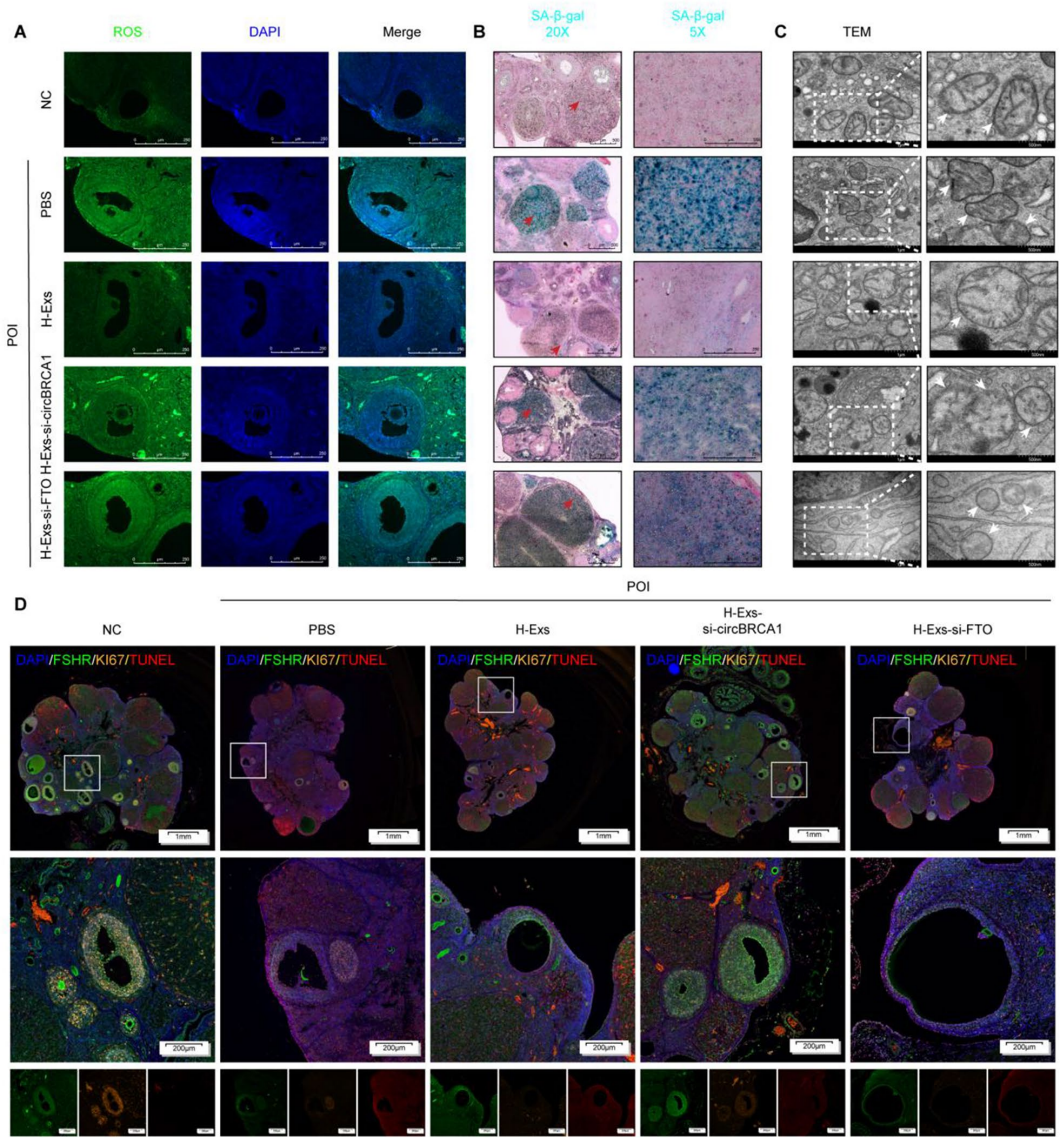
Exosomal circBRCA1 regulates GCs mitochondrial dysfunction and senescence through miR-642a-5p/FOXO1 axis in vivo. **A** The ROS levels were detected by DCFH-DA staining. (scale bar = 50 µm) **B** SA-β-gal staining. (scale bar = 100 µm) **C** Scanning the mitochondria of GCs using TEM. **D** Typical images of multiple fluorescence of rat ovaries. (scale bar = 1 mm)

Subsequently, we assessed the role of H-Exs in the repair of GC_S_ mitochondrial dysfunction and senescence. Compared to PBS, H-Exs reduced ROS accumulation, P21 levels and SA-β-gal activity but elevated SOD_2_ levels (Fig. 7A, B and Supplementary Fig. 8D). In addition, H-Exs improved the mitochondrial morphology of GCs, as evidenced by TEM (Fig. 7C). Moreover, typical multiple fluorescence images showed the colocalization of TUNEL, Ki67 and FSHR positive cells in rat ovaries, indicating enhanced proliferation and attenuated apoptosis of GCs (Fig. 7D). However, these effects were significantly neutralized by circBRCA1/FTO KD (Fig. 7A–D and Supplementary Fig. 8D). In conclusion, circBRCA1 or FTO KD attenuated the therapeutic effect of H-Exs on rats with POI.

**Fig. 8:**
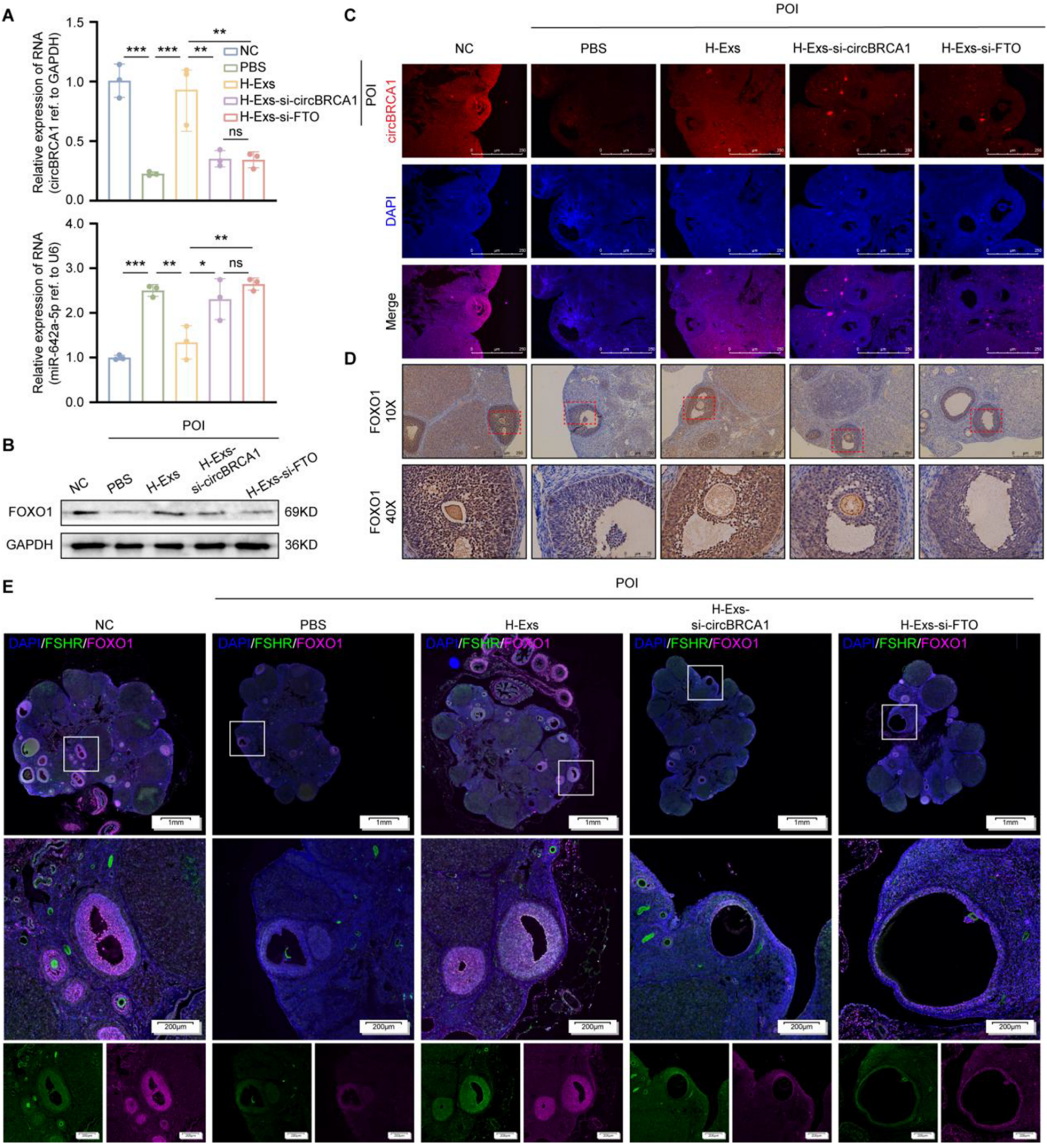
Exosomal circBRCA1 regulates GCs mitochondrial dysfunction and senescence through miR-642a-5p/FOXO1 axis in vivo. **A** Relative expression levels of circBRCA1 and miR-642a-5p in ovaries were determined by RT-qPCR. **B** Western blot analysis revealed the expression levels of FOXO1 in ovaries. **C** Relative expression levels of of circBRCA1 in ovaries were detected by FISH. **D** Immunohistochemistry (IHC) was used to measure the expression of FOXO1 in ovaries. Brown: positive expression of the aimed protein; Blue: nuclear staining. (scale bar = 50 µm) **E** Typical images of multiple fluorescence of rat ovaries. (scale bar = 1 mm)

Compared with those of the NC group, circBRCA1 and FOXO1 levels were significantly reduced in the ovaries of rats with POI and were restored by H-Exs but not H-Exs-si-circBRCA1 or H-Exs-si-FTO treatment. Moreover, circBRCA1 and FOXO1 levels were consistently negatively correlated with miR-642a-5p levels (Fig. 8A, B). The results of IF and IHC showed the same trend consistent with the results of the in vitro study (Fig. 8C–E). Together, these data support the notion that the function of exosomal circBRCA1 in GCs mitochondrial dysfunction and senescence in vivo is likely mediated through the regulation of the miR-642a-5p/FOXO1 axis.

### CircBRCA1 correlates with ovarian reserve function

**Fig. 9:**
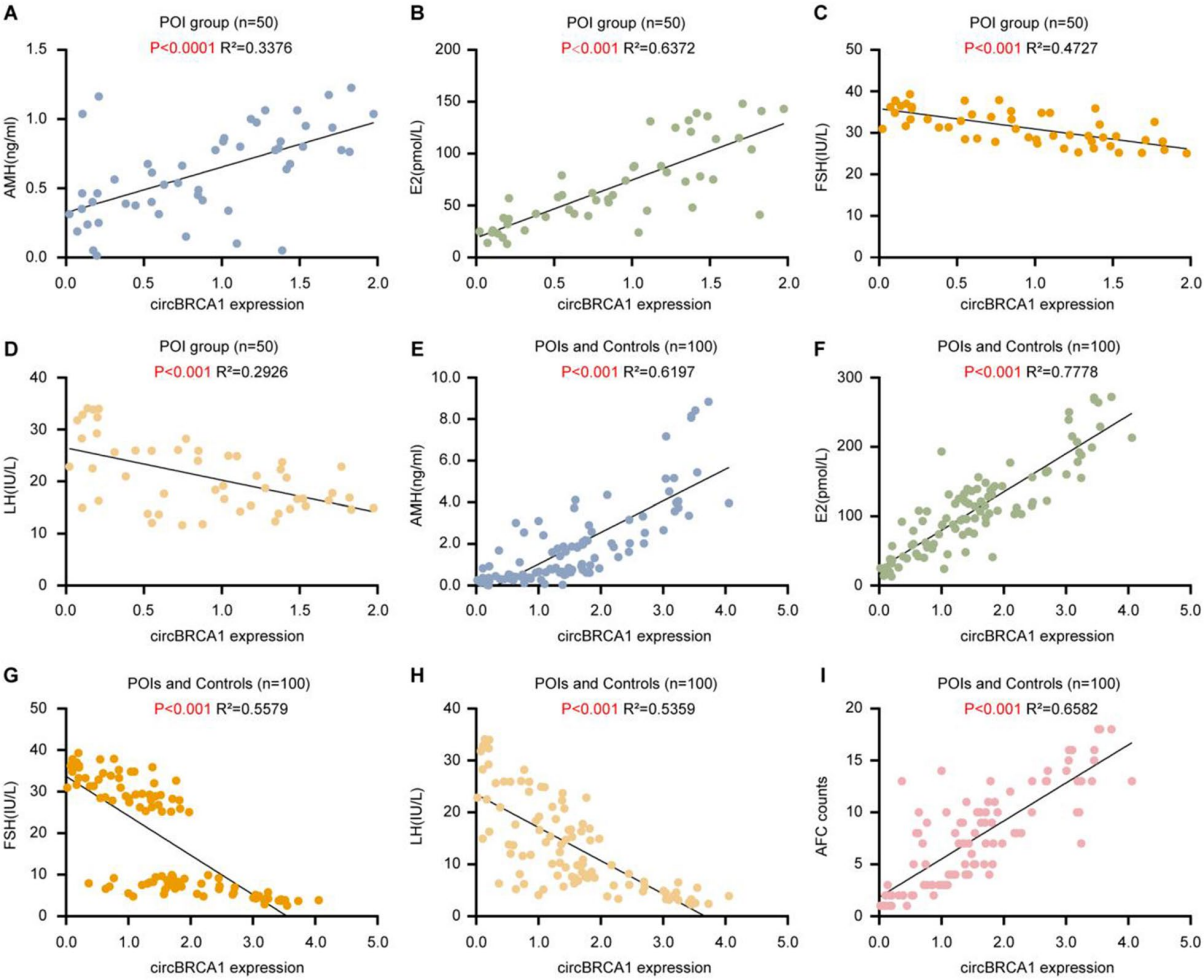
CircBRCA1 is associated with ovarian reserve function. **A**–**D** Correlation of circBRCA1 expression levels in serum with serum estradiol, AMH, FSH, and LH concentrations in POI patients (n = 50) using Pearson correlation analysis. **E**–**H** Correlation of circBRCA1 expression levels in serum with serum E_2_, AMH, FSH, and LH concentrations in all subjects (n = 100) using Pearson correlation analysis. **I** Correlation of circBRCA1 expression levels in serum with AFC in all subjects (n = 100) using Pearson correlation analysis

**Fig. 10:**
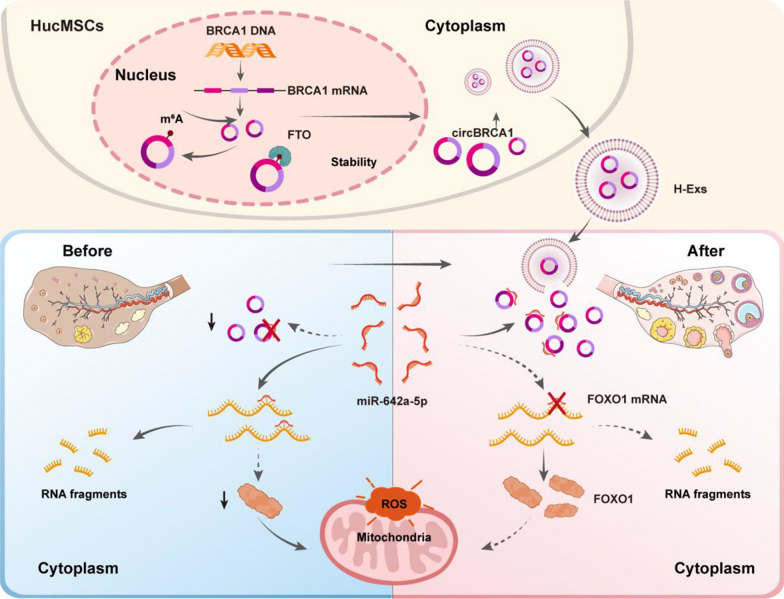
Schematic of the mechanisms involving exosomal circBRCA1 in the regulation of POI

To pursue this putative link between circBRCA1 and ovarian reserve function, we examined the expression level of circBRCA1 in serum and clinical ovarian reserve function indices in 50 patients with POI, and the results showed that circBRCA1 was positively correlated with serum levels of basal E_2_ and AMH (Fig. 9A, B) and antral follicle count (AFC) (Fig. 9I) but negatively correlated with basal FSH and LH in serum (Fig. 9C, D). Similarly, the same trend was obtained by the correlation analysis of all samples (POI and NC groups, n = 100) (Fig. 9E–I). These observations further confirm that circBRCA1 is clinically associated with ovarian reserve function and is expected to be a novel biological marker for POI (Fig. 10)

## Discussion

Excessive oxidative stress in GCs leads to impaired cumulus-oocyte complex maturation, follicle apoptosis, follicle metabolic disturbance or ovarian fibrosis, thereby leading to POI [3, 36]. This study aimed to investigate the therapeutic effects and mechanism of H-Exs on oxidative stress-induced GCs dysfunction. In this molecular event, the demethylase FTO promoted the biological stability of circBRCA1 in HucMSCs. Then, ROS-damaged GCs internalized HucMSC-secreted exosomal circBRCA1, which sponged miR-642a-5p to induce the expression of FOXO1, resulting in improved mitochondrial function and inhibition of cell senescence in GCs. Furthermore, our data indicated that H-Exs could protect against ovarian function decline and fertility in CTX-induced POI rat models, suggesting that H-Exs may be a potential nanotherapeutic agent for the treatment of POI, the pathological basis of which involves GCs dysfunction.

The high-speed development of next-generation sequencing and bioinformatics technology has allowed us to identify the vital roles of circRNAs in different biological processes, especially in carcinogenesis [37]; however, little is known about the functions of circRNAs in the pathology of POI. This study provides the first evidence that circBRCA1 derived from exons 19–21 of the BRCA1 gene contributes to oxidative damage repair in GCs. Analysis of the clinicopathological characteristics of 50 patients with POI revealed that circBRCA1 expression positively correlated with decreased ovarian reserve. Functional analyses further validated the role of circBRCA1 in improving ROS-induced GCs mitochondrial dysfunction and cellular senescence in vivo and in vitro, highlighting the important relationship between circBRCA1 and POI pathogenesis. The functions of circRNAs depend on their intracellular localization, and most cytoplasmic circRNAs act as miRNA sponges, which bind to miRNAs to release their suppression of miRNA-targeted mRNAs. Herein, we identified 9 likely miRNAs with multiple binding sites for the cytoplasmic RNA circBRCA1 by using a computational algorithm. The accurate and authentic interplay between circBRCA1 and miR-642a-5p was corroborated by pulldown and luciferase reporter assays. Further experiments revealed that miR-642a-5p exhibited vital functions in ROS damage repair in GCs. Despite this new finding, the involvement of miR-642a-5p in cell cycle arrest is not a new phenomenon [38, 39].

Subsequently, we found that FOXO1 was a downstream target of miR-642a-5p in GCs. Consistent with the competing endogenous RNA theory, a positive correlation of FOXO1 expression with circBRCA1 expression and a negative correlation of FOXO1 expression with miR-642a-5p expression were detected in GCs of patients with POI. In addition, bioinformatic analysis and functional experiments revealed that circBRCA1 released the suppressive effect of miR-452-5p on FOXO1 expression. Although the role of FOXO1 in POI remains unclear, FOXO1 has been shown to reduce GCs apoptosis during severe hypoxia [40].

M^6^A modification in mRNAs and circRNAs is highly prevalent and can functionally regulate the eukaryotic transcriptome, thereby affecting RNA splicing, export, localization, translation and stability [41]. The present study indicated that FTO was involved in the stability and expression of circRNAs by acting as a demethylase. Consistent with this finding, previous studies illustrated that m^6^A levels were significantly higher in patients with POI than in control subjects and that the decreased mRNA and protein expression levels of FTO may be responsible for the increase in m^6^A in POI [19, 20, 25]. Moreover, FTO has been reported to regulate APOE mRNA stability by reducing m^6^A levels [42]. Herein, FTO decreased the m^6^A level of circBRCA1 but increased circBRCA1 expression. FTO KD in H-Exs, mimicking circBRCA1 silencing effects, attenuated H-Ex-mediated oxidative damage repair and promoted mitochondrial dysfunction and cellular senescence in GCs but limited the specific mechanism by which FTO regulates circBRCA1 stability through m^6^A.

## Conclusion

We identified the protective role of exosomal circBRCA1 in ROS damage and CTX-induced rats with POI by improving mitochondrial function and cell senescence in GCs. Mechanistically, reduced m^6^A levels in the circBRCA1 transcript enhanced its stability and expression, with FTO as a potential eraser in HucMSCs. Exosomal circBRCA1 reduced ROS damage by directly sponging miR-642a-5p to upregulate FOXO1 expression in GCs. In addition, circBRCA1 was downregulated in GCs and serum of patients with POI and was related to reduced ovarian reserve function. Thus, the present study proposes an H-Ex-based and ncRNA-involved approach for the therapeutic intervention of POI and highlights the potential use of circBRCA1 as a biomarker.

### Supplementary Information

Supplementary materials 1.

## Data Availability

Circular RNA expression profiling of human granulosa cells and miRNA profile of the human follicular fluids in young and advanced-aged women were obtained from the GEO database. The GEO accession numbers are GSE97193 and GSE63737.

## Abbreviations

POI: Premature ovarian insufficiency
HucMSCs: Human umbilical cord mesenchymal stem cells
H-Exs: Human umbilical cord mesenchymal stem cell-derived exosomes
GCs: Granulosa cells
circRNAs: Circular RNAs
miRNAs: MicroRNAs
TEM: Transmission electron microscopy
NTA: Nanoparticle tracking analysis
ceRNA: Competitive endogenous RNA
ROS: Reactive oxygen species
SA-β-gal: Senescence-associated β-gal
MMP: Mitochondrial membrane potential
OCR: Oxygen consumption rate
