# Supplementary material for: M6A demethylase FTO-stabilized exosomal circBRCA1 alleviates oxidative stress-induced granulosa cell damage via the miR-642a-5p/FOXO1 axis
Source: J Nanobiotechnology. 2024 Jun 25;22:367. doi: 10.1186/s12951-024-02583-5 (PMC11197183; doi:10.1186/s12951-024-02583-5)
Supplement: Supplementary file 1 — Supplementary materials 1. [file 12951_2024_2583_MOESM1_ESM.pdf]

Fig.S1

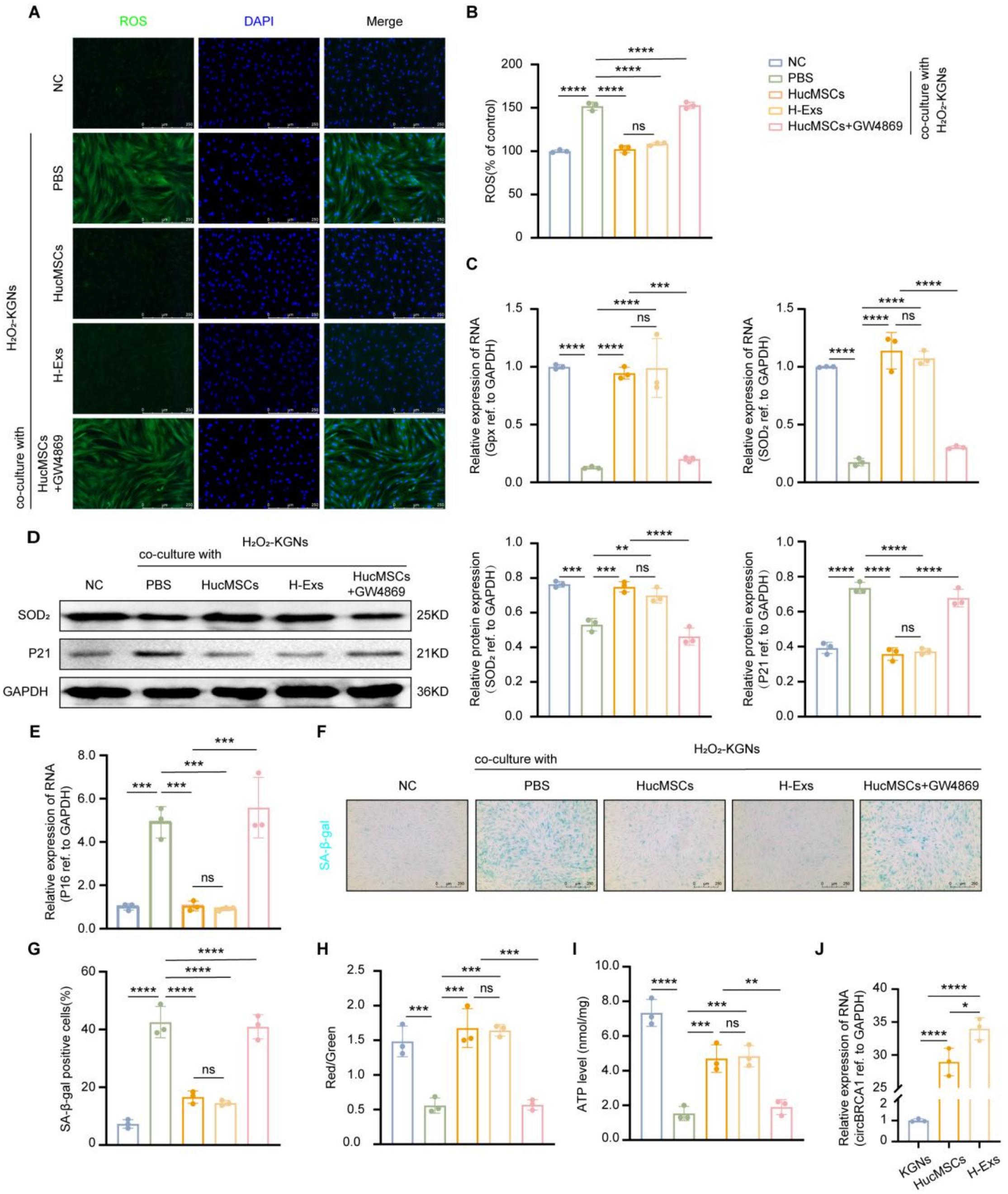

S1. H-Exs repair the oxidative damage in KGNs. A-B The ROS levels were detected by DCFH-DA staining. Green: DCFH-DA staining; Blue: nuclear staining. (scale bar = 50 μm) C Relative expression levels of SOD_2 and Gpx were determined by RT-qPCR. D Western blot analysis revealed the expression levels of SOD_2 and P21 in KGNs. E Relative expression levels of P16 were determined by RT-qPCR. F-G Senescence-associated β-galactosidase (SA-β-gal) staining. Senescent cells were stained blue. (scale bar = 100 μm) H Fluorescence intensity statistics of the JC-1 staining. I Statistical analysis of ATP level. J Relative expression level of circBRCA1 in H_2O_2-KGNs co-culture with PBS and H-Exs.

Fig.S2

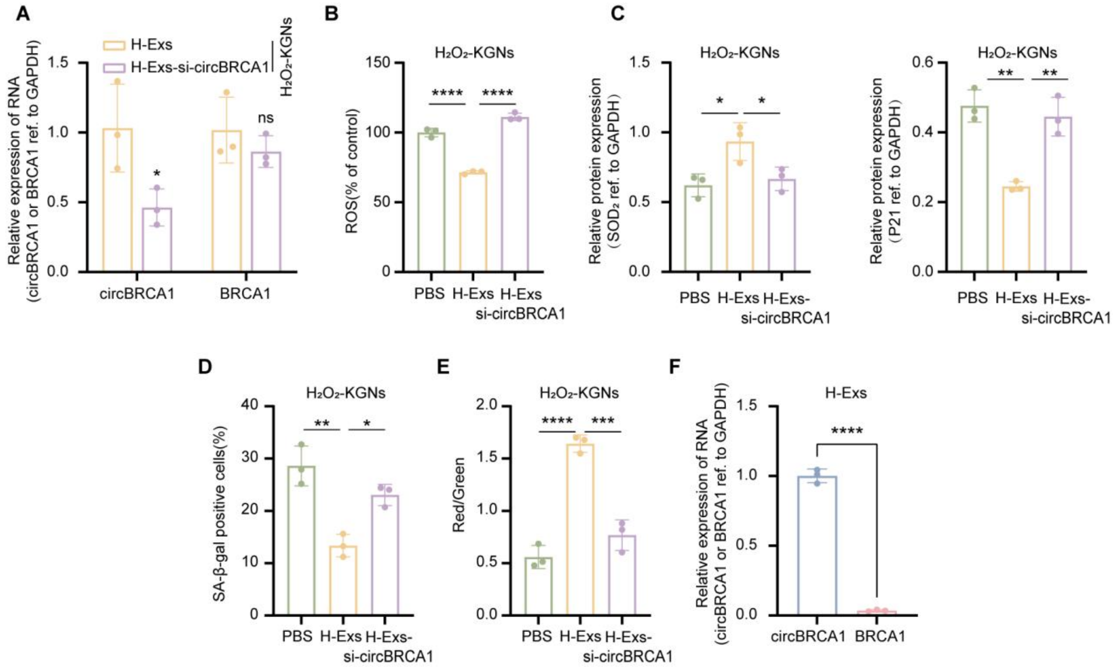

S2. H-Ex-released circBRCA1 is the key for repairing oxidatively damaged KGNs.

A Relative expression levels of circBRCA1 and BRCA1 in KGNs co-cultured with H-Exs and H-Exs-si-circBRCA1 were determined by RT-qPCR. B Fluorescence intensity statistics of DCFH-DA staining. C Statistical analysis of the western blot analysis. D SA-β-gal positive cell counts. E Fluorescence intensity statistics of the JC-1 staining. F Relative expression levels of circBRCA1 and BRCA1 in H-Exs were determined by RT-qPCR.

Fig.S3

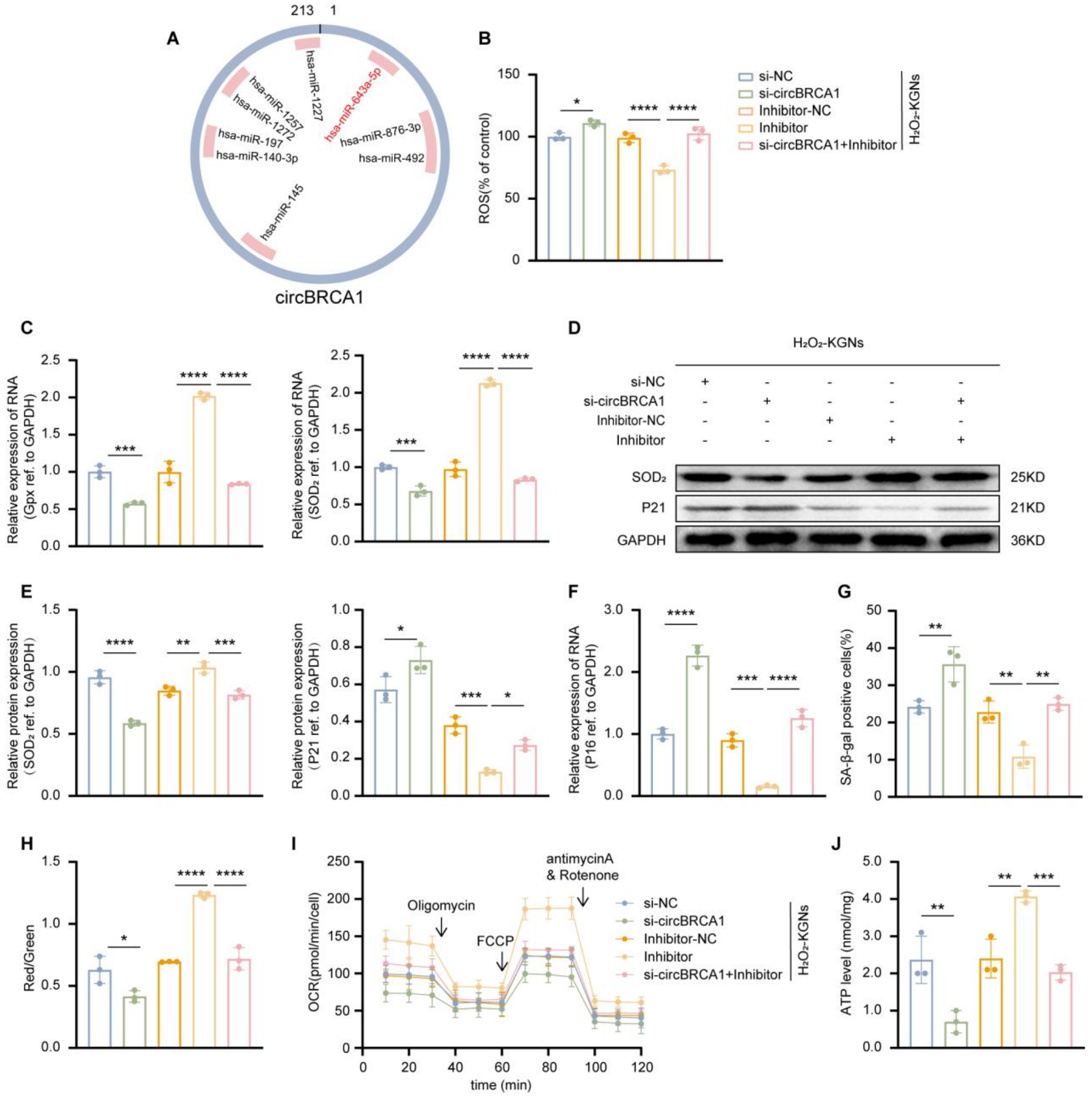

S3.CircBRCA1 acts as a sponge for miR-642a-5p. A The schematic drawing shows the putative binding sites for miRNAs associated with circBRCA1. B Fluorescence intensity statistics of DCFH-DA staining. C Relative expression levels of SOD_2 and Gpx were determined by RT-qPCR. D Western blot analysis revealed the expression levels of SOD_2 and P21 in KGNs. E Statistical analysis of the western blot analysis. F Relative expression levels of P16 were determined by RT-qPCR. G SA- β -gal positive cell counts. H Fluorescence intensity statistics of the JC-1 staining. I Oxygen consumption rate of KGNs. J Statistical analysis of ATP level.

Fig.S4

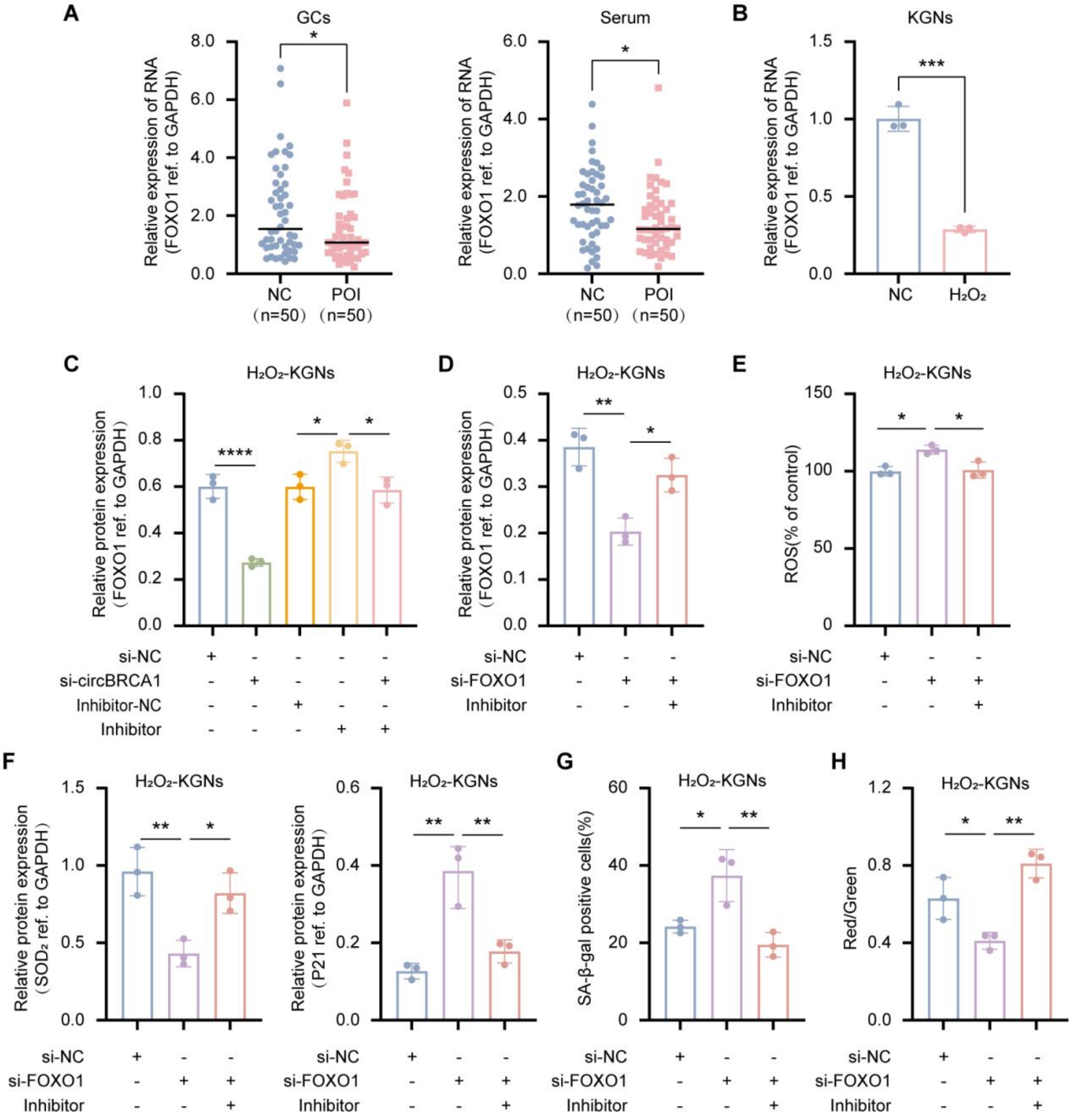

S4. CircBRCA1 upregulates FOXO1 expression by sponging miR-642a-5p. A Relative expression levels of FOXO1 in GCs and serum of patients with normal ovarian function (NC, n=50) and POI patients (POI, n=50) were determined by RT-qPCR. B Relative expression levels of FOXO1 in KGNs and H_2O_2-KGNs were determined by RT-qPCR. C-D Statistical analysis of the western blot analysis. E Fluorescence intensity statistics of DCFH-DA staining. F Statistical analysis of the western blot analysis. G SA- β -gal positive cell counts. H Fluorescence intensity statistics of the JC-1 staining.

Fig.S5

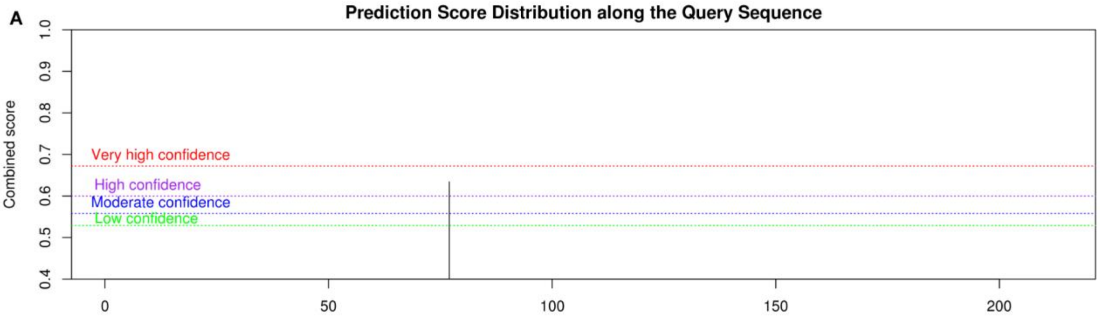

#	Position	Sequence context	Structural context	Local structure visualization	Score(binary)	Score(knn)	Score(spectrum)	Score(combined)	Decision
1	77	AAGCG AGCAA GAGAA UCCCA GGACA GAAAG AUCUU CAGGG GGCUA	N/A	N/A	0.616	0.640	0.658	0.634	m^6A site (**High** confidence)

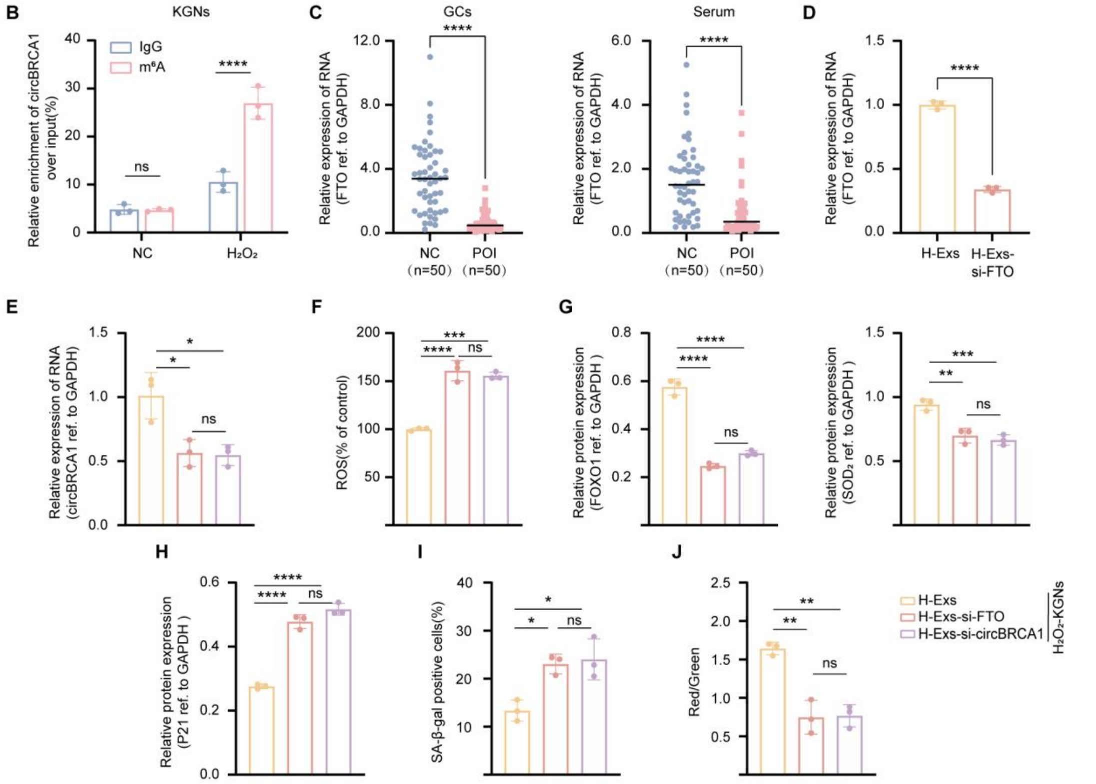

S5. FTO-mediated m^6A demethylation regulates circBRCA1 stability and expression. A Bioinformatics analysis showed that there was a m^6A modification site in circBRCA1. B M^6A RIP assay was performed to detect the enrichment rate of circBRCA1 in KGNs and H_2O_2-KGNs. C Relative expression levels of FTO in GCs and serum of patients with normal ovarian function (NC, n=50) and POI patients (POI, n=50) were determined by RT-qPCR. D Relative expression levels of FTO in H_2O_2-KGNs co-culture with H-Exs and H-Exs-si-FTO were determined by RT-qPCR. E Relative expression levels of circBRCA1 in H_2O_2-KGNs co-culture with H-Exs, H-Exs-si-FTO and H-Exs-si-circBRCA1 were determined by RT-qPCR. F Fluorescence intensity statistics of DCFH-DA staining. G-H Statistical analysis of the western blot analysis. I SA- β -gal positive cell counts. J Fluorescence intensity statistics of the JC-1 staining.

Fig.S6

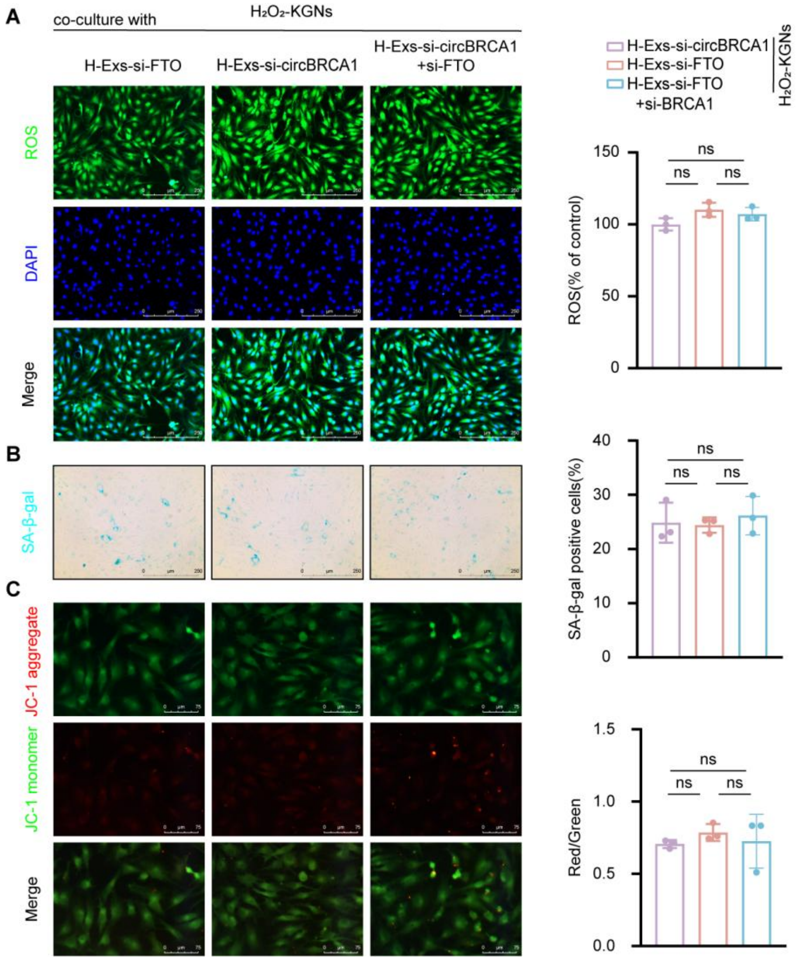

S6. FTO-mediated m^{6}A demethylation regulates circBRCA1 stability and expression. A The ROS levels were detected by DCFH-DA staining. (scale bar = 50 μm) B SA-β-gal staining. (scale bar = 100 μm) C JC-1 staining was used to detect the changes of MMP. (scale bar = 75μm)

Fig.S7

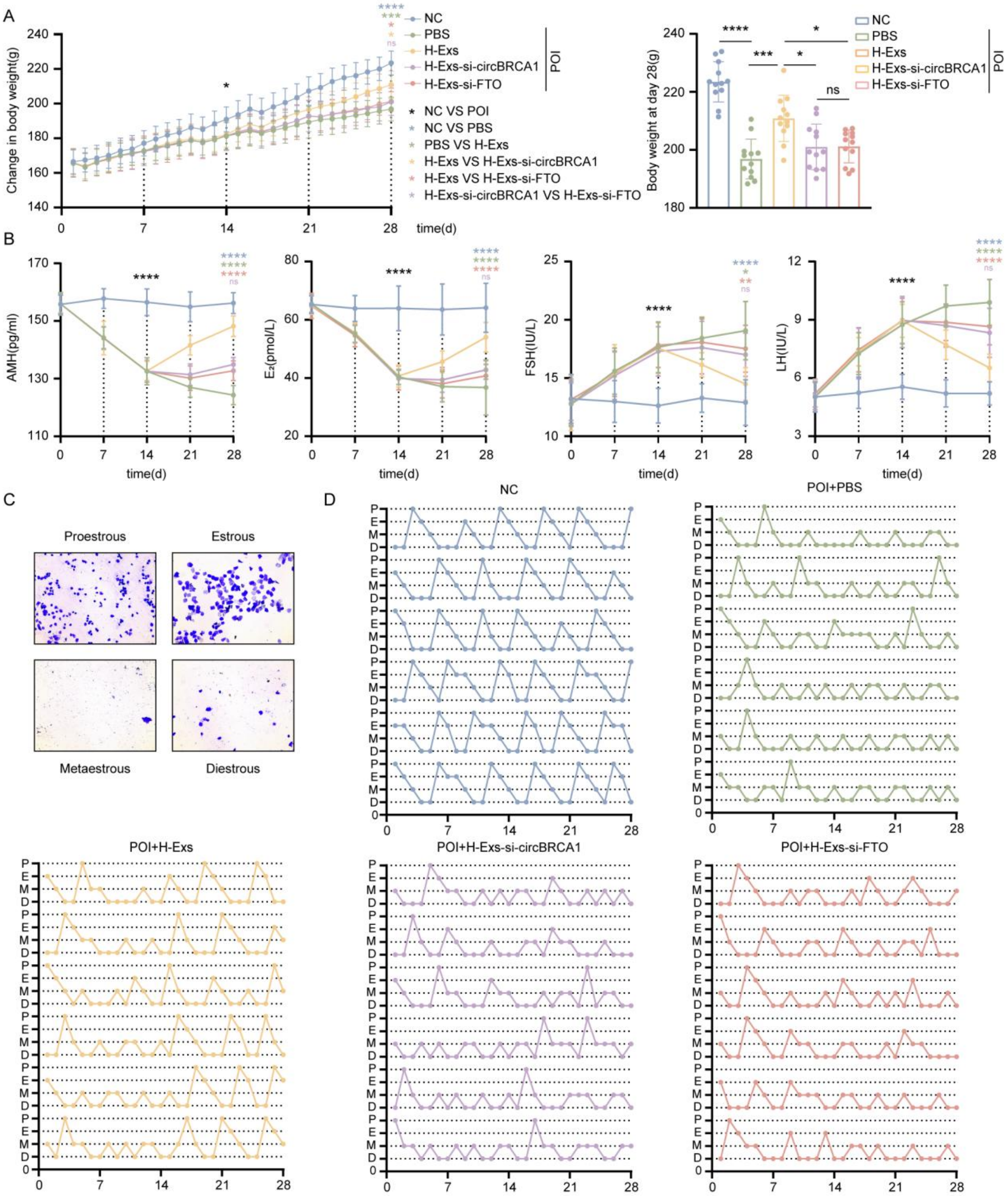

S7. The therapeutic efficacy of exosomal circBRCA1 in treating POI in vivo. A Changes in body weight of rats. B Serum concentrations of AMH, E_2, FSH and LH. C Crystal violet staining of vaginal smears. D The estrous cycle of rats.

Fig.S8

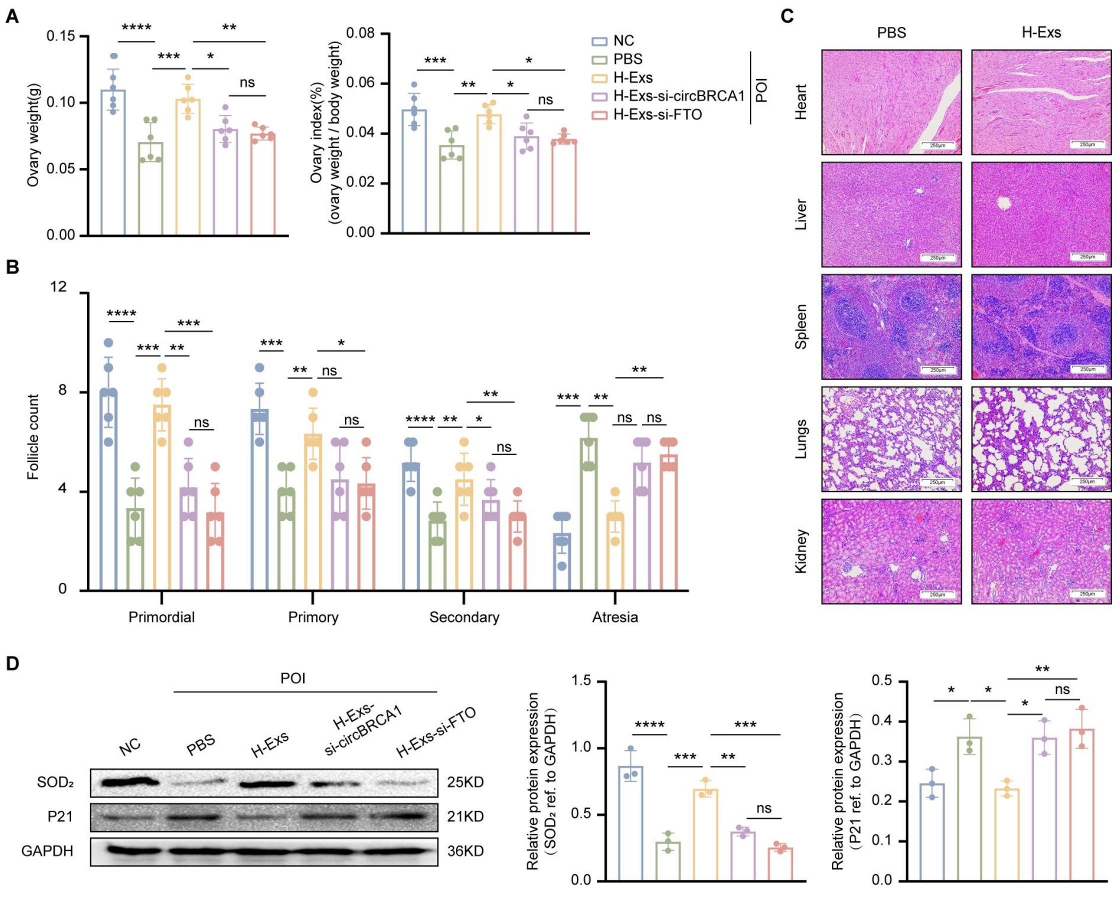

S8. The therapeutic efficacy of exosomal circBRCA1 in treating POI in vivo. A Ovarian weight and ovarian index. B Quantitation of follicle count. C Histopathology of heart, liver, spleen, lung, kidney from rat treated by H-Exs. D Western blot analysis revealed the expression levels of SOD_2 and P21 in ovaries.

Tab.S1 Patients information for this study

Parameters	NC (n=50)	POI (n=50)	*P* value
Age (years)	31.50±5.50	33.00±5.00	*P*=0.458
BMI (kg/m^2)	21.10±2.84	21.53±2.57	*P*=0.513
AMH (ng/mL)	4.94±3.90	0.50±0.49	*P*<0.001
E_2 (pmol/L)	181.00±91.00	80.50±67.50	*P*<0.001
FSH (IU/L)	6.26±3.68	32.19±7.11	*P*<0.001
LH (IU/L)	6.58±4.33	22.55±11.52	*P*<0.001
AFC	12.50±5.50	3.00±2.00	*P*<0.001

Tab. S2 Primers names and sequences

Gene	Forward	Reverse
CircBRCA1	5'-GCTTCTGTGGTGAAGGAGCT-3'	5'-TCTTGCTCGCTTTGGACCTT-3'
Divergent primers	5'-TGGTGCTTCTGTGGTGAAGG-3'	5'-CTCTTGCTCGCTTTGGACCT-3'
Convergent primers	5'-TGAAGTCAGAGGAGATGTGGTCA-3'	5'-GTGCCAAGGGTGAATGATGA-3'
BRCA1	5'-GCCAAAGTAGCTGATGTATTGG-3'	5'-CAGTTACATGGCTTAAGTTGGG-3'
miR-642a-5p	5'-ACGTCCCTCTCCAAATGTGTCTTG-3'	5’-ATCCAGTGCAGGGICCGAGG-3’
FOXO1	5'-TGTCCTACGCCGACCTCATCAC-3'	5'-GCACGCTCTTGACCATCCACTC-3'
FTO	5’- GTTCACAACCTCGGTTTAGTTC-3’	5’- CATCATCATTGTCCACATCGTC-3’
SOD_2	5'-CGCCCTGGAACCTCACATCAAC-3'	5'-AACGCCTCCTGGTACTTCTCCTC-3'
Gpx	5'-AAAGAACTCCTGTCCTCCCACCTC-3'	5'-CCAGCGGATGTCGTGAACCTTC-3'
P16	5’-GTTACGGTCGGAGGCCG-3’	5’-GTGAGAGTGGCGGGGTC-3’
GAPDH	5’-CAGGAGGCATTGCTGATGAT-3’	5’-GAAGGCIGGGGCICATTT-3’
U6	5’-AGAGAAGATTAGCATGGCCCCTG-3’	5’-ATCCAGTGCAGGGICCGAGG-3’
